# Safety and Efficacy of Long-Acting Injectable Agents for HIV-1: Systematic Review and Meta-Analysis

**DOI:** 10.2196/46767

**Published:** 2023-07-27

**Authors:** Wenjing Wang, Shengnan Zhao, Yaxin Wu, Wenshan Duan, Sibo Li, Zhen Li, Caiping Guo, Wen Wang, Tong Zhang, Hao Wu, Xiaojie Huang

**Affiliations:** 1 Clinical and Research Center for Infectious Diseases Beijing Youan Hospital, Capital Medical University Beijing China; 2 Beijing Key Laboratory for HIV/AIDS Research, Clinical and Research Center for Infectious Diseases Beijing Youan Hospital, Capital Medical University Beijing China

**Keywords:** long-acting cabotegravir, CAB-LA, long-acting rilpivirine, RPV-LA, pre-exposure prophylaxis, PrEP, treatment, long-term suppression

## Abstract

**Background:**

HIV-1 infection continues to affect global health. Although antiretrovirals can reduce the viral load or prevent HIV-1 infection, current drugs require daily oral use with a high adherence level. Long-acting antiretrovirals (LA-ARVs) significantly improve medication adherence and are essential for HIV-1 prophylaxis and therapy.

**Objective:**

This study aimed to investigate the safety and efficacy of long-acting cabotegravir (CAB-LA) and long-acting rilpivirine (RPV-LA) in the prevention and treatment of HIV-1 infection.

**Methods:**

PubMed, Embase, and the Cochrane Library were searched for studies from database inception to November 12, 2022. We included studies that reported efficacy and safety data on LA-ARV intervention in people living with HIV and excluded reviews, animal studies, and articles with missing or duplicate data. Virological suppression was defined as plasma viral load <50 copies/mL 6 months after antiviral therapy initiation. We extracted outcomes for analysis and expressed dichotomous data as risk ratios (RRs) and continuous data as mean differences. Depending on the heterogeneity assessment, a fixed- or random-effects model was used for data synthesis. We performed subgroup analyses of the partial safety and efficacy outcomes of CAB-LA+RPV-LA. The protocol was registered with the Open Science Framework.

**Results:**

We included 12 trials comprising 10,957 individuals, of which 7 were prevention trials and 5 were treatment trials. CAB-LA and RPV-LA demonstrated safety profiles comparable with those of the placebo in terms of adverse event–related withdrawal. Moreover, the efficacy data showed that CAB-LA had a better effect on HIV-1 prevention than tenofovir disoproxil fumarate–emtricitabine (17/5161, 0.33% vs 75/5129, 1.46%; RR 0.21, 95% CI 0.07-0.61; *I^2^*=70%). Although CAB-LA+RPV-LA had more drug-related adverse events (556/681, 81.6% vs 37/598, 6.2%; RR 12.50, 95% CI 3.98-39.23; *I^2^*=85%), a mild or moderate injection site reaction was the most common reaction, and its frequency decreased over time. The efficacy of CAB-LA+RPV-LA was comparable with that of daily oral drugs at 48 and 96 weeks (1302/1424, 91.43% vs 915/993, 92.2%; RR 0.99, 95% CI 0.97-1.02; *I^2^*=0%), and a high level of virological suppression of 80.9% (186/230) was maintained even after 5 years of LA-ARV use. Similar efficacy outcomes were observed in both treatment-naive and treatment-experienced patients (849/911, 93.2% vs 615/654, 94%; RR 0.99, 95% CI 0.96-1.02; *I^2^*=0%). According to the questionnaires, more than 85% of people living with HIV favored LA-ARVs.

**Conclusions:**

LA-ARVs showed favorable safety profiles for both the prevention and treatment of HIV-1 infection and were well tolerated. CAB-LA has more satisfactory efficacy than tenofovir disoproxil fumarate–emtricitabine, significantly reducing the rate of HIV-1 infection. CAB-LA+RPV-LA maintains virological suppression for a long time and may be a viable switching strategy with enhanced public health benefits by reducing transmission. However, further trials are required to confirm the efficacy of these drugs.

## Introduction

### Background

HIV-1 infection continues to affect global human health, with an estimated 38.4 million individuals living with HIV-1 by the end of 2021 and 28.7 million accessing antiretroviral therapy (ART) by the end of December 2021 [1]. Despite the enormous progress made in ART, over 5000 people are newly infected with HIV-1 worldwide every day [2]. Studies have shown that ART has demonstrated significant efficacy in limiting HIV-1 viral replication, reducing plasma viral load (VL), and strengthening the immune system. This provides people living with HIV with long-lasting virological suppression [3,4]. The use of ART has the potential to considerably reduce HIV-related morbidity and death, thereby improving the overall health status of people living with HIV and prolonging their life expectancy [5]. Pre-exposure prophylaxis (PrEP) with ART before HIV-1 exposure is a major HIV-1 prevention innovation that can effectively reduce HIV-1 infection rates and provide benefits to populations at high risk for HIV-1 infection [6]. Oral regimens comprising tenofovir disoproxil fumarate–emtricitabine (TDF-FTC) have proven to be effective in preventing HIV-1 infection in high-risk individuals in several clinical trials of HIV-1 PrEP [7,8].

Although antiretrovirals can control the symptoms of people living with HIV, there is no cure for HIV-1 infections. Currently, ART requires lifelong administration, and most antiretrovirals must be taken daily to suppress HIV-1 infection [9,10], which requires a high level of adherence to ART by users [11,12]. people living with HIV or people at high risk of HIV-1 infection face considerable challenges in maintaining the efficacy of ART in the face of the pressure to take daily drugs. In addition, prolonged daily oral medications can lead to treatment fatigue [13,14] coupled with changes in daily lifestyle, stigma associated with long-term medication use, burden of drug use, and multiple social factors [15], all of which can lead to suboptimal adherence and reduce the efficacy of medications or even lead to the emergence of drug-resistant viral variants. Long-acting antiretrovirals (LA-ARVs) have the potential to increase ART adherence, reduce HIV-1 transmission, and achieve public health benefits by decreasing the number of new HIV-1 infections.

The advent of LA-ARVs may overcome several problems associated with the daily use of oral pills and privacy of patients taking drugs. They are used once a month or every 2 months, greatly reducing the frequency and burden of daily medication, eliminating the need to take daily pills [16,17] and improving the convenience of taking medicine while protecting patients’ privacy and reducing the social stigma associated with HIV-1, which is important for people living with HIV and those at high risk of HIV-1 infection. Cabotegravir is a novel integrase inhibitor with acceptable safety and tolerability when administered orally once daily [18]. The nonnucleoside reverse transcriptase inhibitor rilpivirine is administered orally once daily for HIV-1 treatment. LA-ARVs have currently been approved for the treatment and prevention of HIV-1 infection in several countries. In March 2020, ViiV Healthcare announced that Health Canada approved Cabenuva (cabotegravir and rilpivirine extended-release injectable suspensions) for the market. This was the first global approval of Cabenuva as an alternative to existing antiviral regimens for virologically suppressed people living with HIV [19]. The United States Food and Drug Administration has approved Cabenuva as a complete regimen for the treatment of HIV-1 infection in adults with virological suppression on a stable antiretroviral regimen [19] and Apretude (cabotegravir extended-release injectable suspension) for HIV-1 PrEP in high-risk adults and adolescents weighing at least 35 kg [20]. Apretude is also approved as a prophylactic drug in Europe [21], Australia, South Africa [22], Zimbabwe [23], and other countries. In light of the benefits associated with LA-ARVs, there is a growing interest in their development, as well as concerns regarding their potential adverse effects, safety, and efficacy.

### Objectives

Therefore, this meta-analysis aimed to summarize existing trials on LA-ARVs, particularly long-acting cabotegravir (CAB-LA), long-acting rilpivirine (RPV-LA), and CAB-LA+RPV-LA; assess their safety and efficacy; and provide evidence for their widespread use.

## Methods

### Overview

We conducted a meta-analysis using version 6.2 of the Cochrane Handbook for Systematic Reviews of Intervention [24]. We followed the PRISMA (Preferred Reporting Items for Systematic Reviews and Meta-Analyses) guidelines were followed [25] (Multimedia Appendix 1). The protocol was deposited and registered in the Open Science Framework [26].

### Search Strategy

We thoroughly searched the PubMed, Embase, and Cochrane Library databases for eligible articles published from their inception to November 12, 2022. Only articles published in English were included in this study. Search terms included “HIV,” “AIDS,” “long-acting formulations,” “cabotegravir,” “gsk1265744,” and “rilpivirine” (Multimedia Appendix 2). After deleting duplicates, 2 writers independently screened the titles, abstracts, and full-text papers. Disagreements were resolved through dialogue and reaching a consensus.

### Study Eligibility Criteria

The inclusion criteria were as follows: (1) study design, randomized trials; (2) study population, adults aged ≥18 years; (3) intervention, LA-ARVs; and (4) end points, which included safety and efficacy data. For the exclusion criteria, we excluded pregnant or lactating women. We excluded reviews, conference abstracts, case reports, letters, animal studies, irretrievable full-text articles, and studies that shared the same data set. We only included the latest or most detailed data when repeated data were encountered.

Currently, different guidelines have different definitions of HIV-1 virological suppression. According to the World Health Organization guideline [27], we defined virological suppression as a plasma VL <50 copies/mL 6 months after the start of ART.

Drug-related adverse event (AE) and AE-related withdrawals were the primary safety outcomes. The major efficacy outcomes were confirmed with HIV-1 infection for prevention and the percentage of individuals with plasma HIV-1 RNA <50 copies/mL for treatment. Secondary safety outcomes included the proportion of individuals with (1) any AE, (2) AE of grade 3 or higher, (3) injection site reaction (ISR), (4) serious AE (SAE), and (5) death, whereas secondary efficacy outcomes of treatment included (1) changes in CD4^+^T cell counts from baseline, (2) the incidence of confirmed virological failure (VF), and (3) the proportion of resistance-associated mutations (RAMs) in patients who acquired HIV-1 infection or with confirmed virologic failure.

### Data Extraction and Risk of Bias Assessment

A Microsoft Excel spreadsheet was used to extract data from each study. We extracted all relevant data: (1) general information, including the name of the first author and trial, trial number, publication year, and the number of patients; (2) study parameters, including interventions, controls, and study design; and (3) participant characteristics, including age and the proportion of the male population; and (4) outcomes, including safety and efficacy data.

As a means of assessing bias risk, the Cochrane risk-of-bias tool was used to analyze the 5 domains of random sequence generation, allocation concealment, blinding of participants and personnel and outcome assessors, incomplete outcome data, and selective outcome reporting. Findings were classified as low, unclear, or high bias. The 2 reviewers worked separately to extract the data and assess the possibility of bias. Multimedia Appendix 3 [28-42] shows details of the risk of bias assessment.

### Statistical Analysis

RevMan statistical software (version 5.3; The Cochrane Collaboration) was used for statistical analyses and Adobe Illustrator (Adobe) was used for graphical editing and presentation. We used proportion and risk ratio (RR) values to express dichotomous data, whereas for continuous data, we used mean differences. A forest plot was used to estimate cumulative effects. The *I^2^* test revealed statistically significant heterogeneity. Four degrees of heterogeneity were distinguished: 0% to 40%, presumably insignificant; 30% to 60%, medium; 50% to 90%, substantial; and 75% to 100%, high [24]. Subgroup analysis was used to investigate the potential heterogeneity. If applicable, leave-one-out sensitivity analysis was performed to investigate the consistency of the results. If sufficient publications were available, publication bias was investigated using funnel plots and the Egger test.

## Results

### Study Selection and Characteristics

**Figure 1:**
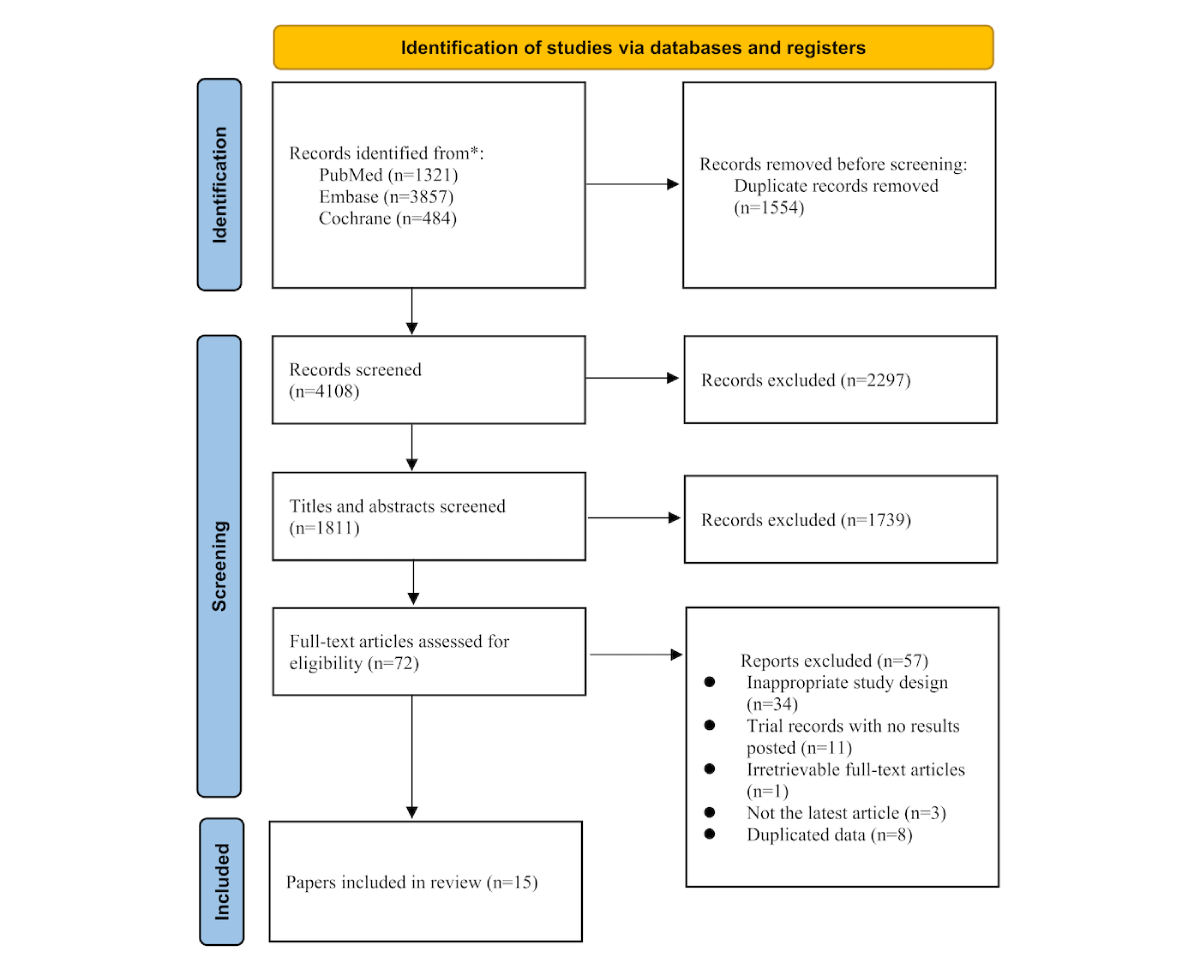
Diagram flow illustrating literature search process. *We searched the database until November 12, 2022.

We obtained 5662 records through an initial literature search, including 1321 in PubMed, 3857 in Embase, and 484 in the Cochrane Library. After removal of duplicates, 4108 studies remained. A total of 72 publications remained after screening the titles and abstracts for full-text inspection. Furthermore, 15 papers comprising 10,957 individuals were ultimately enrolled for data analysis, which contained 12 trials, including 7 trials for prevention (5 on CAB-LA [28-32], 2 on RPV-LA [33,34]), and 5 trials on CAB-LA+RPV-LA [35-42] for treatment. The LATTE-2, ATLAS, and FLAIR treatment trials included one maintenance phase and one extension phase study. Figure 1 illustrates the procedure for conducting the literature search.

CAB-LA was injected intramuscularly and subcutaneously at intervals between 4 and 12 weeks, at dosages ranging from 100 to 800 mg. RPV-LA was injected intramuscularly at doses between 300 and 1200 mg every 4 to 8 weeks. Two doses of CAB-LAs and RPV-LAs were administered intramuscularly. In one case, 400 mg CAB-LA and 600 mg RPV-LA were administered every 4 weeks; in the other case, 600 mg CAB-LA and 900 mg RPV-LA were administered every 8 weeks. Only one CAB-LA trial used both injection methods (intramuscular and subcutaneous). Only intramuscular injection was selected for combined analysis of the data. Table 1 summarizes the characteristics of the included studies.

The bias risk assessment showed that 8 studies [28,29,31-34,36,40] had a low bias risk, whereas 7 open-label trials [30,35,37-39,41,42] with unclear allocation concealment had a high risk of bias (Figures S1 and S2 in Multimedia Appendix 3).

**Table 1:** Characteristics of included studies.

Study (author, year [trial name; NCT ID or Clinicaltrials.gov identifier])			Phase		Masking		Location		Population characteristics		Design		Sample size, n		Age (years)^a^		Male, n (%)
**Prophylaxis**																	
	Landovitz et al [28], 2018 (HPTN 077; NCT02178800)	Ⅱa		Double-blind		Multicenter		Participants were HIV uninfected at screening and at low risk for HIV infection		CAB^b^-LA^c^ IM^d^ 600 mg Q8W^e^ vs placebo		89 (69I^f^, 20C^g^)		31 (24-37)		29 (33)	
	Landovitz et al [28], 2018 (HPTN 077; NCT02178800)	Ⅱa		Double-blind		Multicenter		Participants were HIV uninfected at screening and at low risk for HIV infection		CAB-LA IM 800 mg Q12W^h^ vs placebo		110 (82I, 28C)		33 (25-42)		38 (35)	
	Markowitz et al [29], 2017 (ECLAIR; NCT02076178)	Ⅱa		Double-blind		Multicenter		Participants were male at birth, HIV uninfected, and reported having at least one casual se partner in the past 24 mo		CAB-LA IM 800 mg Q12W vs placebo		127 (106I, 21C)		31 (20-61)		127 (100)	
	Spreen et al [30], 2014 (NCT01756131)	Ⅰ		Open-label		Single-center		Healthy volunteers		CAB-LA IM 100, 200, 200×2, 400, 400×2 mg single-dose vs placebo		72 (58I, 14C)		35.1 (10.4)		39 (54.2)	
	Landovitz et al [31], 2021 (HPTN 083; NCT02720094)	Ⅱb-Ⅲ		Double-blind		Multicenter		Adults had a negative HIV serological test at enrollment and had an undetectable blood HIV RNA viral load within 14 days before trial entry		CAB-LA IM 600 mg Q8w with TDF^i^-FTC^j^ placebo QD^k^ vs TDF-FTC QD with CAB-LA placebo IM Q8w		4570 (2283I, 2287C)		26 (22-32)		3992 (87.4)	
	Delany-Moretlwe et al [32], 2022 (HPTN 084; NCT03164564)	Ⅲ		Double-blind		Multicenter		Female reported at least 2 episodes of vaginal intercourse in the previous 30 days were at risk of HIV infection based on an HIV risk score		CAB-LA IM 600 mg Q8w with TDF-FTC placebo QD vs TDF-FTC QD with CAB-LA placebo IM Q8w		3224 (1614I, 1610C)		25 (22-30)		0 (0)	
	Verloes et al [33] 2015, (NCT01031589)	Ⅰ		Open-label; double-blind		Single-center		Healthy volunteers		RPV-LA IM 300,600 mg single-dose; RPV-LA IM 1200 and 600 and 600 mg Q4w^m^ vs placebo		11; 8 (6I, 2C)		47 (31-58); 47 (31-58)		6 (31.6)	
	Bekker et al [34], 2020 (HPTN 076; NTC02165202)	Ⅱ		Double-blind		Multicenter		Healthy, sexually active, low-risk, HIV-uninfected women		RPV^l^-LA IM 1200 mg Q8W vs placebo		136 (91I, 45C)		31 (25-38)		0 (0)	
**Treatment**																	
	Margolis et al [35] 2017, (LATTE-2; NCT02120352)	Ⅱb		Open-label		Multicenter		Treatment-naive adults with HIV-1 who were given either CAB 30 mg PO^n^+ABC^o^/3TC^p^ 600/300 mg PO QD for 20 weeks and who had a VL^q^<50 copies/mL		CAB-LA IM 400 mg+RPV-LA IM 600 mg Q4W or CAB-LA IM 600 mg+RPV-LA IM 900 mg Q8W vs CAB PO 30 mg + ABC PO 600 mg + 3TC PO 300 mg QD		286 (115I,115I, 56C)		35 (19-64)		262 (92)	
	Smith et al [36], 2021 (LATTE-2 extension phase; NCT02120352)	Ⅱb		Open-label		Multicenter		Adults completing 96 weeks of LATTE-2 enter an extension phase		CAB-LA IM 400 mg+RPV-LA IM 600 mg Q4W; CAB-LA IM 600 mg+RPV-LA IM 900 mg Q8W. Optimized loading dose (100 weeks) followed by CAB-LA IM 400 mg+RPV-LA IM 600 mg Q4W; Optimized loading dose (100 weeks and 104 weeks) followed by CAB-LA IM 600 mg+RPV-LA IM 900 mg Q8W		115; 115; 10; 34		36 (19-62); 34 (20-64); 41 (21-56); 36 (19-56)		109 (95); 107 (93); 8 (80); 28 (82)	
	Orkin et al [37], 2021 (FLAIR; NCT02938520)	Ⅲ		Open-label		Multicenter		Treatment-naive adults with HIV-1 who were given DTG^q^/ABC/3TC PO 50/600/300 mg QD for 20 weeks and who had a VL<50 copies/mL		CAB PO 30 mg+RPV PO 25 mg QD for 4 weeks, followed by CAB-LA; IM 600 mg+RPV-LA IM 900 mg, then CAB-LA IM 400 mg+RPV-LA IM 600 mg Q4W for 100 weeks vs DTG PO 50 mg + ABC PO 600 mg + 3TC PO 300 mg QD for 100 weeks		566 (283I, 283C)		34 (29-43)		439 (78)	
	Orkin et al [38], 2021 (FLAIR extension phase; NCT02938520)	Ⅲ		Open-label		Multicenter		Adults completing 100 weeks of ATLAS enter an extension phase		Switched from CAB 30 mg+RPV 25 mg QD to CAB-LA+RPV-LA (direct-to-injection group); switched from CAB 30 mg+RPV 25 mg QD to CAB-LA+RPV-LA (oral lead-in group); continued the long-acting regimen		111; 121; 283		36 (30-45); 38 (31-46); 34 (29-42)		24 (22); 27 (22); 63 (22)	
	Swindells et al [39], 2020 (ATLAS; NCT02951052)	Ⅲ		Open-label		Multicenter		Adults with HIV-1 and had a VL<50 copies/mL for ≧6 months while taking PI-, NNRTI-, or INSTI-based regimen with a two-NRTI backbone		CAB 30 mg+RPV 25 mg QD for 4 weeks, followed by CAB-LA IM 600 mg+RPV-LA IM 900 mg, then CAB-LA IM 400 mg+RPV-LA IM 600 mg Q4W for 52 weeks vs PI-, NNRTI-, or INSTI-based QD for 52 weeks		616 (308I, 308C)		42 (18-82)		413 (67)	
	Swindells et al [40], 2022 (ATLAS extension phase; NCT02951052)	Ⅲ		Open-label		Multicenter		Adults completing 52 weeks of ATLAS enter an extension phase		CAB-LA IM 400 mg+RPV-LA IM 600 mg Q4W vs switched from CAB 30 mg+RPV 25 mg QD to CAB-LA IM 400 mg+RPV-LA IM 600 mg Q4W		52 (23, 29)		—^r^		—	
	Jaeger et al [41], 2021 (ATLAS-2M; NCT03299049)	Ⅲb		Open-label		Multicenter		Parents from ATLAS with a VL<50 copies/mL at screening and additional adults with HIV-1 and a VL<50 copies/mL for ≥6 months while taking standard oral ART		CAB-LA IM 400 mg+RPV-LA IM 600 mg Q4W; CAB-LA IM 600 mg+RPV-LA IM 900 mg Q8W		523; 522		42 (34-50)		765 (73)	
	Mills et al [42], 2021 (POLAR; NCT03639311)	Ⅱb		Open-label		Multicenter		Adults with HIV-1 who had a VL<50 copies/mL and completed at least 300 weeks of the LATTE study		CAB-LA IM 600 mg+RPV-LA IM 900 mg Q8W for 48 weeks vs DTG PO 50 mg + RPV PO 25 mg QD for 48 weeks		97 (90I, 7C)		41 (25-63)		92 (94.8)	

^a^Unless otherwise stated, age is presented as mean (SD) or median (IQR).

^b^CAB: cabotegravir.

^c^LA: long-acting.

^d^IM: intramuscular.

^e^Q8W: every 8 weeks.

^f^I: intervention group.

^g^C: control group.

^h^Q12W: every 12 weeks.

^i^TDF: tenofovir.

^j^FTC: emtricitabine.

^k^QD: daily.

^l^RPV: rilpivirine.

^m^Q4W: every 4 week.

^n^PO: per os.

^q^DTG: dolutegravir.

^o^ABC: abacavir.

^p^3TC: lamivudine.

^q^VL: viral load.

^r^Not available.

### PrEP Medication

#### Safety

**Figure 2:**
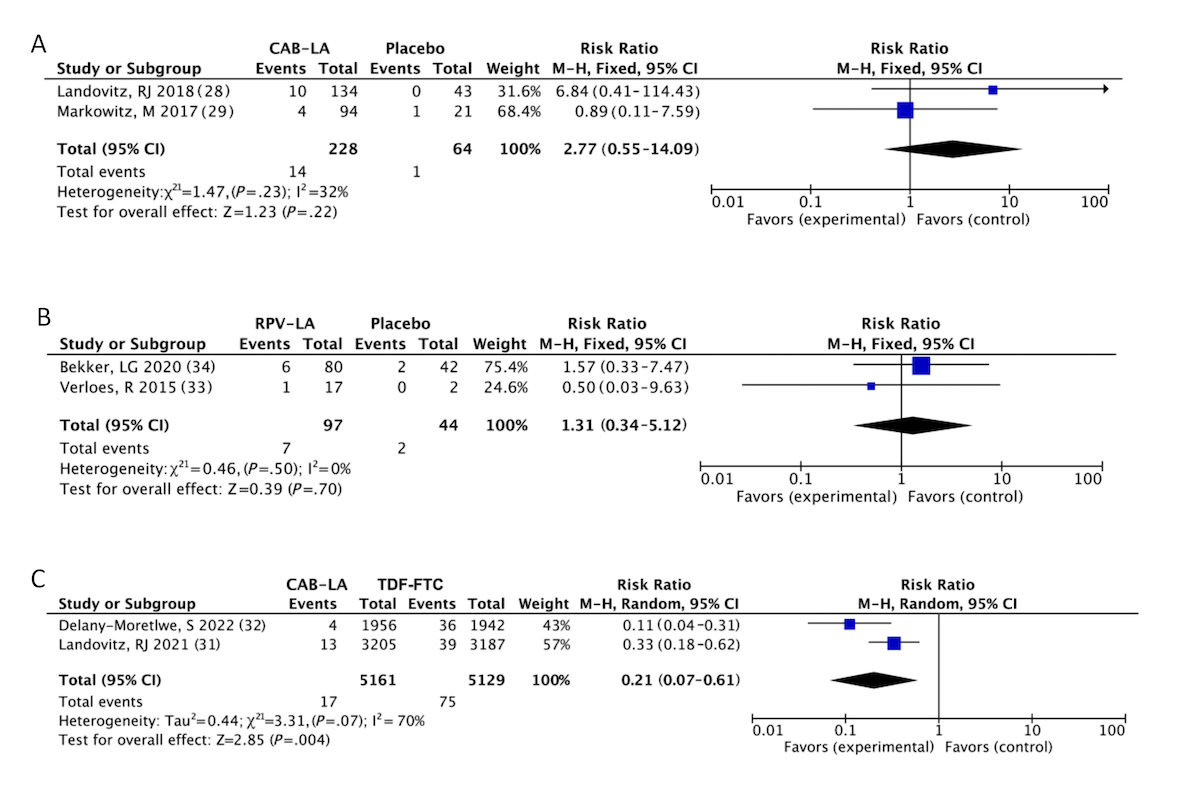
Meta-analyses on safety and efficacy profiles of CAB-LA and RPV-LA: (A) AE-related withdrawals: CAB-LA versus placebo [28,29], (B) AE-related withdrawal: RPV-LA versus placebo [33,34], and (C) confirmed HIV-1 infection: CAB-LA versus TDF-FTC [31,32]. AE: adverse event; CAB-LA: long-acting cabotegravir; RPV-LA: long-acting rilpivirine; TDF-FTC: tenofovir disoproxil fumarate–emtricitabine.

For CAB-LA, 3 of the 5 trials were placebo controls and 2 were TDF-FTC controls. Compared with the placebo, the frequency of AE-related withdrawals (14/228, 6.1% vs 1/64, 2%; RR 2.77, 95% CI 0.55-14.09; *I^2^*=32%; Figure 2A) was similar, as was any AE or SAE (Figures S1A and S1B in Multimedia Appendix 4 [28-40,42]). In contrast, there was high heterogeneity for ISR (165/268, 61.6% vs 15/72, 21%; RR 3.38, 95% CI 0.79-14.44; *I^2^*=80%; Figure S1C in Multimedia Appendix 4). Because of the lack of studies, we could not determine the origin of heterogeneity.

Compared with daily oral TDF-FTC, there was no apparent difference in AE of grade 3 or higher (1003/3894, 25.76% vs 1047/3892, 26.9%; RR 0.96, 95% CI 0.89-1.03; *I^2^*=0%; Figure S1D in Multimedia Appendix 4) and SAE (153/3894, 3.93% vs 154/3892, 3.96%; RR 0.99, 95% CI 0.80-1.24; *I^2^*=0%; Figure S1E in Multimedia Appendix 4). In comparison, CAB-LA had more ISRs (2301/3799, 60.57% vs 815/3798, 21.46%; RR 3.03, 95% CI 2.27-4.04; *I^2^*=91%; Figure S1F in Multimedia Appendix 4) than TDF-FTC. Although ISR was more frequent in the intervention group, the most frequently reported side effect was injection site pain. ISR is often mild to moderate in severity and its frequency gradually diminishes.

Similarly, RPV-LA was well tolerated. For the primary outcome, the occurrence of AE-related withdrawal (7/97, 7% vs 2/44, 5%; RR 1.31, 95% CI 0.34-5.12; *I^2^*=0%; Figure 2B) did not differ between the 2 groups. Analyses of the SAE and ISR (Figures S1G and S1H in Multimedia Appendix 4) showed no obvious differences.

#### Efficacy

The ECLAIR trial [29] revealed 1 proven HIV-1 infection in the cabotegravir group throughout the follow-up period and 1 confirmed HIV-1 infection in the placebo group within the injection phase. In addition to TDF-FTC’s reduction in HIV-1 infection by TDF-FTC, CAB-LA also reduced the rate by 76% (17/5161, 0.33% vs 75/5129, 1.46%; RR 0.21, 95% CI 0.07-0.61; *I^2^*=70%; Figure 2C). One of the 13 participants who acquired HIV-1 infection after enrollment in the CAB-LA group of the HIV Prevention Trials Network (HPTN) 083 trial [31] was reclassified as having a baseline infection. Only 4 infections occurred with regular CAB-LA injections and 2 had drug-resistant mutations.

In HPTN 083 trial [31], 86% of the TDF-FTC group participants maintained plasma concentrations over the lower limit of quantification (0.31 ng/mL), which showed high adherence to oral drugs. In the HPTN 084 trial [32], 2 of the 4 patients with infections in the CAB-LA group had undetectable plasma cabotegravir concentrations, one had delayed cabotegravir injections, and one had baseline infection. None of the infections occurred with on-time injections. The TDF-FTC group documented poor or nonadherence in 35 of the 36 infections, and TDF concentrations greater than 0.31 ng per mL were observed in 55.91% (1084/1939) of participants.

For RPV-LA, the HPTN 076 trial [34] reported that one placebo participant developed HIV-1 infection during the injection period. A total of 67.9% (76/112) of the participants strongly agreed to use RPV-LA at the time of their last injection visit, and 88% stated that they would use it in the future. The efficacy and safety results of the prophylactic drugs are detailed in Multimedia Appendix 5 [28-34]. The RAMs in the people living with HIV are detailed in Multimedia Appendix 6 [28-42].

### Treatment

#### Safety

**Figure 3:**
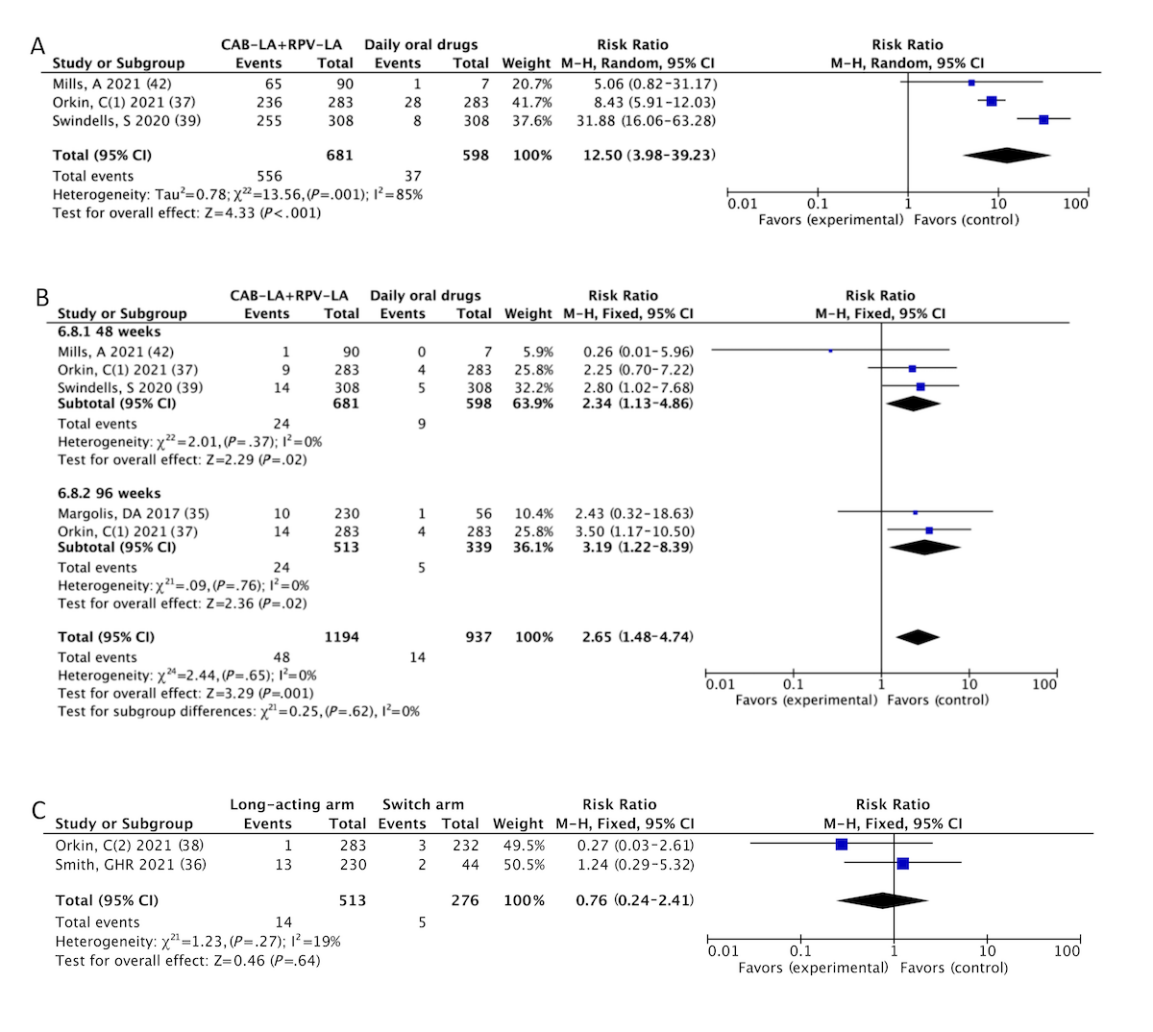
Meta-analyses on safety profiles of CAB-LA+RPV-LA: (A) drug-related AE: CAB-LA+RPV-LA versus daily oral drugs [37,39,42], (B) AE-related withdrawal: CAB-LA+RPV-LA versus daily oral drugs [35,37,39,42], and (C) AE-related withdrawal: long-acting arm versus switch arm [36,38]. AE: adverse event; CAB-LA: long-acting cabotegravir; RPV-LA: long-acting rilpivirine.

Table 2 presents the results for the CAB-LA+RPV-LA. Data analysis revealed that the AE rate of CAB-LA+RPV-LA was higher than that of the daily oral drugs. The incidence of drug-related AE (556/681, 81.6% vs 37/598, 6.2%; RR 12.50, 95% CI 3.98-39.23; *I^2^*=85%; Figure 3A) at 48 weeks in CAB-LA+RPV-LA group was higher than that in the daily oral group, so were the AE-related withdrawal (48/1194, 4.02% vs 14/937, 1.5%; RR 2.65, 95% CI 1.48-4.74; *I^2^*=0%; Figure 3B) at 48 weeks and 96 weeks, any AE (647/681, 95% vs 448/598, 74.9%; RR 1.27, 95% CI 1.12-1.44; *I^2^*=74%; Figure S2A in Multimedia Appendix 4), and AE of grade 3 or higher (75/681, 11% vs 34/598, 5.7%; RR 1.93, 95% CI 1.30-2.87; I^2^=5%; Figure S2B in Multimedia Appendix 4).

When ISR was excluded, drug-related AE after excluding ISR (167/591, 28.3% vs 36/591, 6.1%; RR 5.41, 95% CI 1.35-21.64; *I^2^*=91%; Figure S2C in Multimedia Appendix 4) and AE of grade 3 or higher after excluding ISR (47/591, 8% vs 34/591, 5.8%; RR 1.38, 95% CI 0.90-2.12; *I^2^*=45%; Figure S2D in Multimedia Appendix 4) were reanalyzed. There was no obvious difference in the SAE (Figure S2E in Multimedia Appendix 4). No deaths occurred because of the drugs used.

When patients in the LATTE-2, ATLAS, and FLAIR trials entered the extension phase [31,32,35], people on daily oral medication switched to CAB-LA+RPV-LA, and the long-acting group continued their previous treatment. No statistically significant differences were observed between the groups. The incidence of AE-related withdrawal (14/513, 2.7% vs 5/276, 1.8%; RR 0.76, 95% CI 0.24-2.41; *I^2^*=19%; Figure 3C), any AE (506/513, 98.6% vs 246/276, 89.1%; RR 1.06, 95% CI 0.89-1.25; *I^2^*=97%; Figure S3A in Multimedia Appendix 4), and SAE (39/513, 7.6% vs 16/276, 5.8%; RR 0.82, 95% CI 0.46-1.47; *I^2^*=0%; Figure S3B in Multimedia Appendix 4) in extension phase did not differ significantly between the long-acting arm and the switch arm.

**Table 2:** Safety and efficacy results of CAB^a^-LA^b^+RPV^c^-LA (all results are expressed in terms of frequency (n/N) unless otherwise stated).

Trial name	Design	Safety								Efficacy		
		Any AE^d^	Drug-related AE	AE of grade 3 or higher	SAE^e^	ISR^f^	AE withdrawal	Death	HIV-1 RNA level<50 copies/mL		Median change from baseline in CD4^+^ lymphocyte count-per mm^3^ (n)	Confirmed VF^g^
LATTE-2 (96 weeks)	CAB-LA IM^h^ 400 mg+RPV-LA IM 600 mg Q4W^i^; CAB-LA IM 600 mg+RPV-LA IM 900 mg Q8W^j^; CAB PO^k^ 30 mg+ABC^l^ PO 600 mg + 3TC^m^ PO 300 mg QD^n^	N/A^o^	N/A	N/A	11/115; 11/115; 7/56	112/115; 110/115	8/115; 2/115; 1/56	N/A	100/115; 108/115; 47/56		226 (IQR 145 to 393) (100); 239 (IQR 111 to 359) (109); 317 (IQR 214 to 505) (47)	0; 2/115; 1/56
LATTE-2 extension phase (256 weeks)	CAB-LA IM 400 mg+RPV-LA IM 600 mg Q4W; CAB-LA IM 600 mg+RPV-LA IM 900 mg Q8W; Optimized loading dose (100 weeks) followed by CAB-LA IM 400 mg+RPV-LA IM 600 mg Q4W; Optimized loading dose (100 weeks and 104 weeks) followed by CAB-LA IM 600 mg+RPV-LA IM 900 mg Q8W	115/115; 115/115; 10/10; 34/34	NR^p^	38/115; 39/115; 3/10; 7/34	27/115; 25/115; 1/10; 6/34	N/A	20/115; 3/115; 1/10; 1/34	3/115; 0; 0; 0	85/115; 101/115; 9/10; 32/34		396 (SD 294) (85); 326 (SD 218) (102); 211 (SD 318) (9); −14 (SD 319) (32)	NR
FLAIR (48 weeks)	CAB-LA IM 400 mg+RPV-LA IM 600 mg Q4W; DTG^q^ PO 50 mg + ABC PO 600 mg + 3TC PO 300 mg QD	267/283; 225/283	236/283; 28/283	31/283; 11/283	18/283; 12/283	N/A	9/283; 4/283	0; 0	265/283; 264/283		NR	4/283; 3/283
FLAIR (96 weeks)	CAB-LA IM 400 mg+RPV-LA IM 600 mg Q4W; DTG PO 50 mg + ABC PO 600 mg + 3TC PO 300 mg QD	274/283; 242/283	246/283; 33/283	40/283; 16/283	24/283; 22/283	N/A	14/283; 4/283	0; 0	245/283; 253/283		57 (IQR −43 to 181) (246); 109.5 (IQR 18 to 228) (254)	4/283; 4/283
FLAIR (124 weeks)	DTI group (after 24 weeks of CAB+RPV); OLI group (after 24 weeks of CAB+RPV); Randomly assigned long-acting arm (after 124 weeks of CAB+RPV)	102/111; 100/121; 276/283	86/111; 79/121; 248/283	5/111; 9/121; 49/283	4/111; 5/121; 33/283	NR	1/111; 2/121; 15/283	0; 0; 0	110/111; 113/121; 227/283		NR	N/A
ATLAS (48 weeks)	CAB-LA IM 400 mg+RPV-LA IM 600 mg Q4W; PI-, NNRTI-, or INSTI-based QD	294/308; 220/308	255/308; 8/308	35/308; 23/308	13/308; 14/308	N/A	14/308; 5/308	0; 1/308	285/308; 294/308		4.0 (IQR −536 to 801) (308); 13.5 (IQR −1043 to 521) (308)	3/308; 4/308
ATLAS (96 weeks)	CAB-LA IM 400 mg+RPV-LA IM 600 mg Q4W; Switched from CAB 30 mg+RPV 25 mg QD to CAB-LA IM 400 mg+RPV-LA IM 600 mg Q4W	N/A	N/A	N/A	N/A	N/A	N/A	0; 0	23/23; 28/29		−5.7 (SD 167.6) (23); −33.6 (SD 145.3) (29)	0; 0
ATLAS-2M (96 weeks)	CAB-LA IM 400 mg+RPV-LA IM 600 mg Q4W; CAB-LA IM 600 mg+RPV-LA IM 900 mg Q8W	499/523; 488/522	413/523; 415/522	NR	28/523; 33/522	400/517; 412/516	19/523; 18/522	1/523; 1/522	472/523; 475/522		NR	2/523; 9/522
POLAR (52 weeks)	CAB-LA IM 600 mg+RPV-LA IM 900 mg Q8W; DTG PO 50 mg + RPV PO 25 mg QD	86/90; 3/7	65/90; 1/7	9/90; 0/7	5/90; 1/7	N/A	1/90; 0/7	NR	88/90; 7/7		−12.5 (IQR −138 to 71) (90); −68 (IQR −152 to 152) (7)	0; 0

^a^CAB: cabotegravir.

^b^LA: long-acting.

^c^RPV: rilpivirine.

^d^AE: adverse event.

^e^SAE: serious adverse event.

^f^ISR: injection-site reaction.

^g^VF: virological failure.

^h^IM: intramuscular.

^i^Q4W: every 4 weeks.

^j^Q8W: every 8 weeks.

^k^PO: per os.

^l^ABC: abacavir.

^m^3TC: lamivudine.

^n^QD: daily.

^o^N/A: not available.

^p^NR: not reported.

^q^DTG: dolutegravir.

#### Efficacy

**Figure 4:**
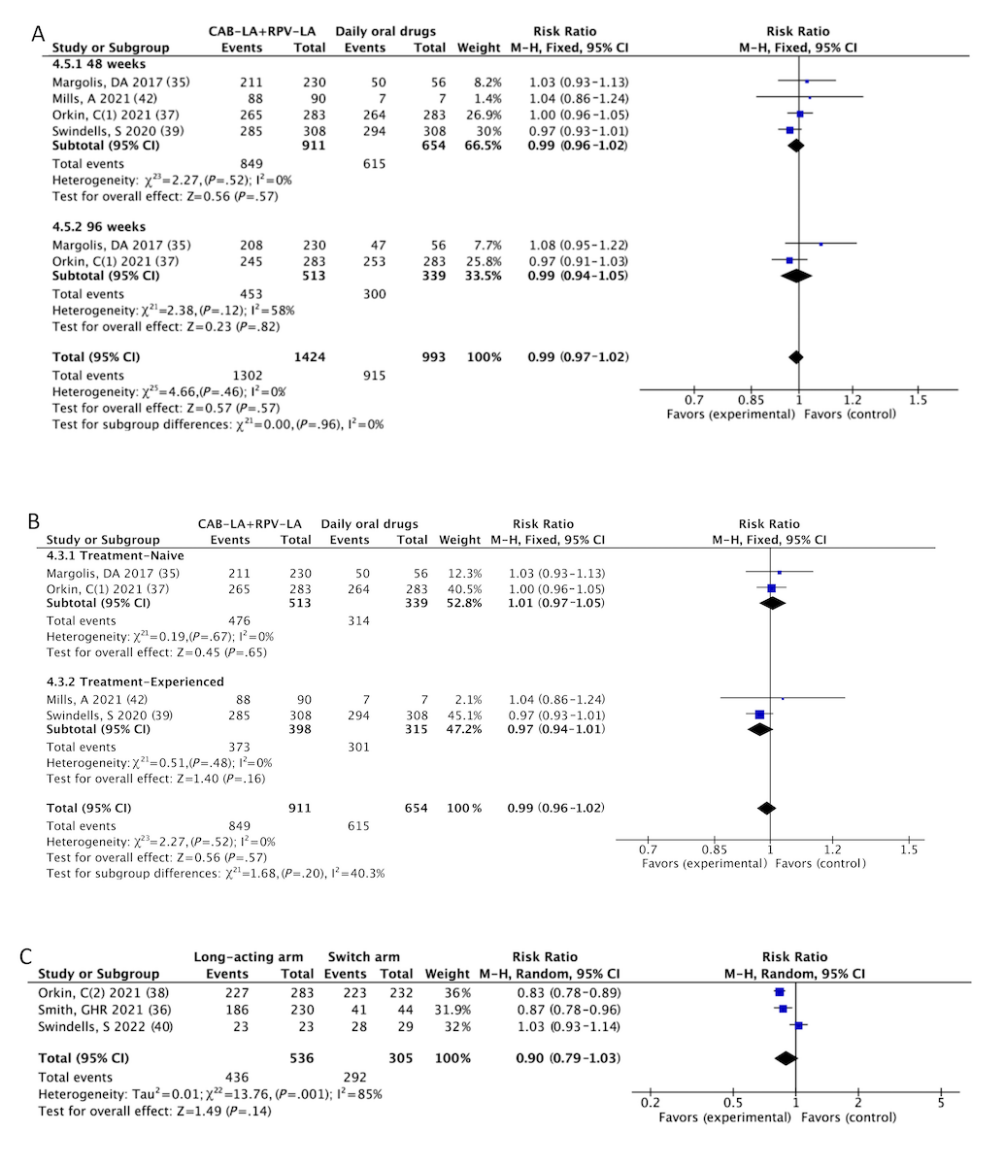
Meta-analyses on efficacy profiles of CAB-LA+RPV-LA: (A) plasma HIV-1 RNA less than 50 copies/mL: CAB-LA+RPV-LA versus daily oral drugs [35,37,39,42], (B) plasma HIV-1 RNA less than 50 copies/mL: CAB-LA+RPV-LA versus daily oral drugs [35,37,39,42], and (C) plasma HIV-1 RNA less than 50 copies/mL: long-acting arm versus switch arm [36,38,40]. CAB-LA: long-acting cabotegravir; RPV-LA: long-acting rilpivirine.

In terms of efficacy, we performed a subgroup analysis of the percentage of individuals with plasma HIV-1 RNA levels <50 copies/mL (1302/1424, 91.43% vs 915/993, 92.2%; RR 0.99, 95% CI 0.97-1.02; *I^2^*=0%; Figure 4A) and the incidence of confirmed VF (13/1104, 1.18% vs 12/930, 1.3%; RR 0.93, 95% CI 0.43-2.04; *I^2^*=0%; Figure S2F in Multimedia Appendix 4) at 48 weeks and 96 weeks. Similarly, when we grouped the treatment-naive patients in the study with treatment-experienced patients (849/911, 93.2% vs 615/654, 94%; RR 0.99, 95% CI 0.96-1.02; *I^2^*=0%; Figure 4B), there were no statistically significant differences in the efficacy outcomes.

We summarized and analyzed the results of RAMs in patients with VF during treatment. The results showed no statistical difference between the CAB-LA+RPV-LA group and the daily oral drug group in terms of RAMs associated with integrase inhibitors (6/9, 67% vs 0/9, 0%; RR 4.64, 95% CI 1.00-21.62; *I^2^*=0%; Figure S1A in Multimedia Appendix 6) or nonnucleoside reverse transcriptase inhibitors (7/9, 78% vs 3/9, 34%; RR 2.12, 95% CI 0.90-4.97; *I^2^*=0%; Figure S2B in Multimedia Appendix 6). In addition, a bar chart of the number of RAMs occurring between groups is presented in Figure S3 in Multimedia Appendix 6.

For patients in the extension phase, there was no significant difference in the percentage of infected people with plasma HIV-1 RNA less than 50 copies/mL (436/536, 81.3% vs 292/305, 95.7%; RR 0.90, 95% CI 0.79-1.03; *I^2^*=85%; Figure 4C) between those who continued LA-ARVs and those who switched to LA-ARVs. This was the change from the baseline in CD4^+^ T cell counts (Figure S3C in Multimedia Appendix 4). Only one participant in the switch arm reported confirmed VF during the extension phase of the FLAIR trial. The 96-week results of the ATLAS-2M [41] corroborated that 90.62% (947/1045) of individuals maintained plasma HIV-1 RNA levels of less than 50 copies/mL. Compared with daily oral ART, patient satisfaction levels with CAB-LA+RPV-LA were high.

Most participants preferred the long-acting regimen; after all injections, 99% (206/208) of the long-acting group in the LATTE-2 trial [35] expressed satisfaction with their current ongoing therapy. In the ATLAS and POLAR trials [39,42], 97.4% (266/273) and 87.5% (77/88) of respondents in the long-acting group, who filled out the questionnaires, said they were inclined to use CAB-LA+RPV-LA instead of daily oral drugs. In the FLAIR extension phase [38], we found that 4 people living with HIV using CAB-LA+RPV-LA for 124 weeks had RAMs, 3 of which had been detected at 96 weeks of the FLAIR trial. The detailed resistance data are provided in Multimedia Appendix 6.

## Discussion

### Principal Findings

To the best of our knowledge, this meta-analysis is the first to assess the safety and efficacy of LA-ARVs in people living with HIV, as well as the advantages of LA-ARVs as a switching strategy. We found a previously published article on LA-ARVs that focused on exploring the safety and pharmacokinetic profiles of 2 drugs, CAB-LA and RPV-LA, for use as PrEP in the prevention of HIV-1 infection [43]. We added newly updated articles on this premise, including data from the HPTN 083 [31] and HPTN 084 [32] trials. The CAB-LA was examined and described in greater detail. In addition, we not only focused on prophylactic drugs but also compared the existing daily antiretrovirals with all existing trials of the combination of LA-ARVs for treating HIV-1 infection. We collected study data with a long follow-up period, and in addition to the results from the maintenance phase of treatment, we collected data within the extension phase for CAB-LA+RPV-LA, providing information on the efficacy beyond 96 weeks and even up to 5 years. To provide more comprehensive evidence for the use of LA-ARVs, we extracted and analyzed the safety and efficacy data, adherence, and patient satisfaction. The availability of LA-ARVs is important for HIV-1 prevention and treatment and for reducing HIV-1 transmission. LA-ARVs with longer dose intervals significantly improve medication adherence by reducing the burden of daily dosing [44], protecting patient privacy, and reducing the stigma associated with HIV-1 infection.

This analysis suggests that participants have a favorable safety profile for LA-ARVs in terms of either prevention or treatment. LA-ARVs have also shown better efficacy than daily oral drugs; however, fewer trials and more studies are required to validate their efficacy. First of all, all participants tolerated the prophylactic drugs, CAB-LA and RPV-LA. Although we observed differences in drug-related AE between the 2 groups in the ECLAIR trial [29], the majority were classified as grade 1 or 2, had little effect on the participant, and were not life-threatening. CAB-LA had more ISRs than oral TDF-FTC owing to the injection of CAB-LA placebo in the control group, which had less of an effect on the organism. Most ISRs in the long-acting group were mild or moderate with a low frequency, and a few patients dropped out of the trial due to ISR. Therefore, CAB-LAs have a good safety profile as a prophylactic agent. CAB-LA is more effective than TDF-FTC in preventing HIV-1 infection and can effectively lower the incidence of HIV-1 infection. Adherence has been shown to have a substantial effect on drug efficacy. The efficacy results from the HPTN 084 trial were comparable with those of the HPTN 083 trial; however, participants in the HPTN 084 trial had lower adherence to the drug, resulting in a higher infection rate in the oral drug group. Second, most infections that occurred in the CAB-LA group in both trials [31,32] were due to low or undetectable plasma concentrations, which were the result of participants not injecting the dose on time or the infection was occurring during the oral induction phase. A combined analysis of the efficacy results from the 2 trials revealed a high degree of heterogeneity, which may be attributable to the variance in the enrolled populations. The statistically significant difference in efficacy in the HPTN 083 trial also demonstrated the superiority of CAB-LA over TDF-FTC. Therefore, cabotegravir is superior to TDF-FTC in preventing HIV-1 infection and has adherence advantages.

However, we observed 4 breakthrough infections in the CAB-LA group in the HPTN 083 trial. Researchers believe that this could be due to several factors, such as low concentrations of cabotegravir in the plasma or rectal tissue, delayed detection of HIV-1 infection, and drug resistance [45]. As mentioned in the package inserts of Apretude, drugs that might significantly reduce cabotegravir’s plasma concentration should be avoided. Therefore, to conduct a more thorough investigation of the efficacy of CAB-LA as PrEP, additional data, including pharmacokinetics and other characteristics, are needed, and in the future, additional trials will be done in multiple enrollment populations.

CAB-LA+RPV-LA also had a better safety profile in treating people living with HIV, although patients experienced more AEs (any AE, drug-related AE, AE-related withdrawals, and AE of grade 3 or higher) at the primary end point time of 48 or 96 weeks than those on daily oral drugs. Firstly, similar to CAB-LA, ISR was the most prevalent AE, predominantly mild or moderate, and its frequency decreased as the study progressed. A few patients dropped out of the trials because of ISR, and most participants generally accepted the overall ISR. We reanalyzed the results of drug-related AE and AE of grade ≥3 after excluding ISR. The results demonstrated no statistically significant difference between the 2 groups in terms of AE of grade 3 or higher after excluding ISR. In contrast, most drug-related AEs, excluding ISR, were fever, which is a subjective symptom of patients and may affect the experimental results. In addition, we observed a lower incidence of ISR in the extension phase than in the maintenance phase, which could be attributed to tolerance to long-term injections.

Second, most control groups might have continued their current daily oral drugs, whereas the intervention groups underwent the oral induction phase or switching therapy, which could have led to more AE in the long-acting group. This possibility was consistent with the findings of a previous switch study [46] that the consequences of starting a new treatment instead of continuing the same treatment may lead to an increase in AE. Although patients who received LA-ARVs experienced more AEs, cabotegravir plus rilpivirine was the most popular regimen. Even in participants who switched from daily oral drugs to CAB+RPV, the safety results were not significantly different from those who used CAB-LA+RPV-LA for a long time. Most participants preferred the cabotegravir plus rilpivirine therapy and presented higher levels of satisfaction.

There was no significant difference between CAB-LA+RPV-LA and daily oral drugs in terms of the primary efficacy end point, and CAB-LA+RPV-LA maintained virological suppression in patients well, even in the extension phase, with the advantage of a long injection interval, which proved that CAB-LA+RPV-LA was superior to daily oral agents. In addition, analysis of data from patients treated with CAB-LA+RPV-LA for more than 2 years revealed that the HIV-1 viral suppression rate was maintained at a high level, even in patients who received LA-ARVs for 5 years [36], indicating that CAB-LA+RPV-LA had the capacity to suppress VL for a long time. This finding lays the foundation for the long-lasting effects of LA-ARVs. However, subgroup analysis of the efficacy outcomes of treatment-naive and treatment-experienced patients who participated in the study revealed heterogeneity between subgroups, indicating that variances in the enrolled population may have influenced the trial results. We observed that the enrolled studies maintained virological suppression before receiving LA-ARVs or after the oral induction phase. According to the guidelines [47], if patients achieve virological suppression of their current ART, arbitrary regimen adjustments are not recommended but may be considered in certain specific situations, such as simplifying the regimen by reducing the number of pills and frequency of administration to facilitate a smooth drug switch. In these trials [36,38,40], patients who smoothly switched from daily oral drugs to LA-ARVs were able to maintain high levels of virological suppression, with no statistically significant differences in efficacy compared with patients on LA-ARVs, which provides support for individuals who are currently receiving oral therapy and wish to switch to LA-ARVs. If LA-ARVs are popularized in the future, CAB-LA + RPV-LA may be a good switching strategy for individuals who use daily oral drugs for long periods and maintain virological suppression.

We observed VFs in both the long-acting arm and daily oral drug arm and analyzed their RAMs. There were no statistically significant differences between the groups; however, because of the limited number of studies and participants, no definitive conclusions could be drawn. Most patients in the long-acting group continued to receive LA-ARVs and had a higher predilection for LA-ARVs than daily oral drugs. Thus, CAB-LA+RPV-LA, which greatly improved patient adherence, provided a more convenient dosing regimen and optimized the daily oral regimen in people living with HIV, who achieved and effectively maintained virological suppression. It performed well in terms of safety and tolerance of drug use in infected patients and could be an effective and promising treatment or switching strategy. However, additional trials are required to confirm the results of our study.

Our study is significant for the promotion of LA-ARVs. The emergence of LA-ARVs has made the prevention and treatment of AIDS easier. It reduces the burden on people living with HIV of different genders, avoids stigmatization associated with daily oral drug treatment, and improves the correct use and compliance of doses. This is particularly important to ensure the effectiveness of pre-exposure prevention measures. Improving the prevention and treatment of HIV-1 will reduce its transmission and contribute to reducing the prevalence of new HIV-1 infections. The United Nations Programme on HIV/AIDS has called for these life-changing injectable drugs to be quickly available, affordable, and fairly distributed to those who need them the most worldwide [48]. This requires more regional and national regulators to quickly approve and adopt a series of measures to reduce sales prices. National HIV-1 prevention programs need to develop LA-ARVs promotion plans and help health systems and communities to prepare for the use of this new drug.

### Limitations

This review had several limitations. First, because the results are presented in several formats, it is impossible to integrate data for analysis such as the median form. Second, owing to the relatively low number of trials, we could not perform a publication bias analysis, and the lack of trials on RPV-LA and efficacy data may lead to potential bias. Third, so far, LA-ARVs have been studied mainly in high-income countries, and the cost of cabotegravir and rilpivirine may also be a major obstacle to their supply in low- and middle-income countries. Therefore, more research is needed to determine the efficacy, acceptability, and economic burden of LA-ARVs in different income groups, particularly in low-income and middle-income countries. Finally, we retained only the original articles and trials, discarded conferences, and the resulting data that appeared at the conference. This could have led to the omission of valid data and weakened the persuasiveness of the results.

### Conclusions

In conclusion, our findings support the safety and efficacy of CAB-LA, RPV-LA, and CAB-LA+RPV-LA, which are effective and well-tolerated monthly or bimonthly injections that can provide PrEP to people at risk of HIV-1 infection, reduce the rate of HIV-1 infection, and provide a more convenient treatment option for people living with HIV. CAB-LA+RPV-LA maintained virological suppression of HIV-1 for approximately 2 years, with good efficacy even after 5 years of treatment. Similarly, effective virological suppression was maintained after switching from daily oral drugs to LA-ARVs in people living with HIV who successfully achieved virological suppression. In summary, LA-ARVs have shown good safety and tolerability in dosing, can effectively improve patient adherence, protect patient privacy, and can be a promising treatment or an alternative to oral ART. Currently, multiple LA-ARVs have been evaluated in clinical trials, including injectables, rings, and implants, such as PrEP and subcutaneous injections, for treating HIV-1 infection [49]. For example, a phase 2/3 CAPELLA study on lenacapavir confirmed a high rate of virological suppression in patients with multidrug resistance [50], and islatravir has also been confirmed for drug safety in a phase I trial [51]. Therefore, research and development of LA-ARVs is tremendously advantageous for HIV-1 prevention and treatment. Given the lack of such studies, further investigation of the efficacy of RPV-LAs is required.

## Appendix

Multimedia Appendix 1 PRISMA (Preferred Reporting Items for Systematic Reviews and Meta-Analyses) checklists.

Multimedia Appendix 2 Search strategy.

Multimedia Appendix 3 Risk of Bias for meta-analyses.

Multimedia Appendix 4 Forest plots for the outcomes.

Multimedia Appendix 5 Safety and efficacy profiles of long-acting antiretroviral drugs for prophylaxis.

Multimedia Appendix 6 Drug resistance data.

## Abbreviations

AE: adverse event
ART: antiretroviral therapy
CAB-LA: long-acting cabotegravir
ISR: injection site reaction
LA-ARV: long-acting antiretroviral
PrEP: pre-exposure prophylaxis
PRISMA: Preferred Reporting Items for Systematic Reviews and Meta-Analyses
RAM: resistance-associated mutation
RPV-LA: long-acting rilpivirine
RR: risk ratio
SAE: serious adverse event
TDF-FTC: tenofovir disoproxil fumarate–emtricitabine
VF: virological failure
VL: viral load

## Data Availability

All data generated or analyzed in this study are publicly available and are included in this published article.
