# Supplementary material for: Safety and Efficacy of Long-Acting Injectable Agents for HIV-1: Systematic Review and Meta-Analysis
Source: JMIR Public Health Surveill. 2023 Jul 27;9:e46767. doi: 10.2196/46767 (PMC10415942; doi:10.2196/46767)
Supplement: Multimedia Appendix 2 [file publichealth_v9i1e46767_app2.docx]

**Multimedia Appendix 2. Search strategy**

| Databse | Keywords |
| --- | --- |
| PubMed (1321) | ("hiv"[MeSH Terms] OR "hiv"[All Fields] OR ("acquired immunodeficiency syndrome"[MeSH Terms] OR ("acquired"[All Fields] AND "immunodeficiency"[All Fields] AND "syndrome"[All Fields]) OR "acquired immunodeficiency syndrome"[All Fields] OR "aids"[All Fields])) AND (("long-acting"[All Fields] AND ("drug compounding"[MeSH Terms] OR ("drug"[All Fields] AND "compounding"[All Fields]) OR "drug compounding"[All Fields] OR "formulation"[All Fields] OR "formulate"[All Fields] OR "formulated"[All Fields] OR "formulates"[All Fields] OR "formulating"[All Fields] OR "formulation s"[All Fields] OR "formulations"[All Fields] OR "formulative"[All Fields] OR "formulator"[All Fields] OR "formulators"[All Fields])) OR ("islatravir"[Supplementary Concept] OR "islatravir"[All Fields]) OR ("islatravir"[Supplementary Concept] OR "islatravir"[All Fields] OR "mk 8591"[All Fields]) OR ("cabotegravir"[Supplementary Concept] OR "cabotegravir"[All Fields] OR "gsk1265744"[All Fields]) OR ("cabotegravir"[Supplementary Concept] OR "cabotegravir"[All Fields]) OR "lenacapavir"[All Fields] OR "gs6207"[All Fields] OR ("rilpivirine"[MeSH Terms] OR "rilpivirine"[All Fields])) |
| Embase (3857) | #1‘hiv’/exp OR hiv  #2 aids  #3 islatravir  #4‘mk 8591’  #5 gsk1265744  #6 cabotegravir  #7 lenacapavir  #8 gs6207  #9 rilpivirine  #10‘long acting’AND formulations  #11 #1 OR #2  #12 #3 OR #4 OR #5 OR #6 OR #7 OR #8 OR #9 OR #10  #13 #11 AND #12 |
| Cochrane Library (484) | (hiv or aids):ti,ab,kw AND (long-acting formulations or islatravir or mk-8591 or gsk1265744 or cabotegravir or lenacapavir or gs6207 or rilpivirine ):ti,ab,kw |
