# Supplementary material for: Safety and Efficacy of Long-Acting Injectable Agents for HIV-1: Systematic Review and Meta-Analysis
Source: JMIR Public Health Surveill. 2023 Jul 27;9:e46767. doi: 10.2196/46767 (PMC10415942; doi:10.2196/46767)
Supplement: Multimedia Appendix 3 [file publichealth_v9i1e46767_app3.docx]

**Multimedia Appendix 3. Risk of Bias for meta-analyses**

**
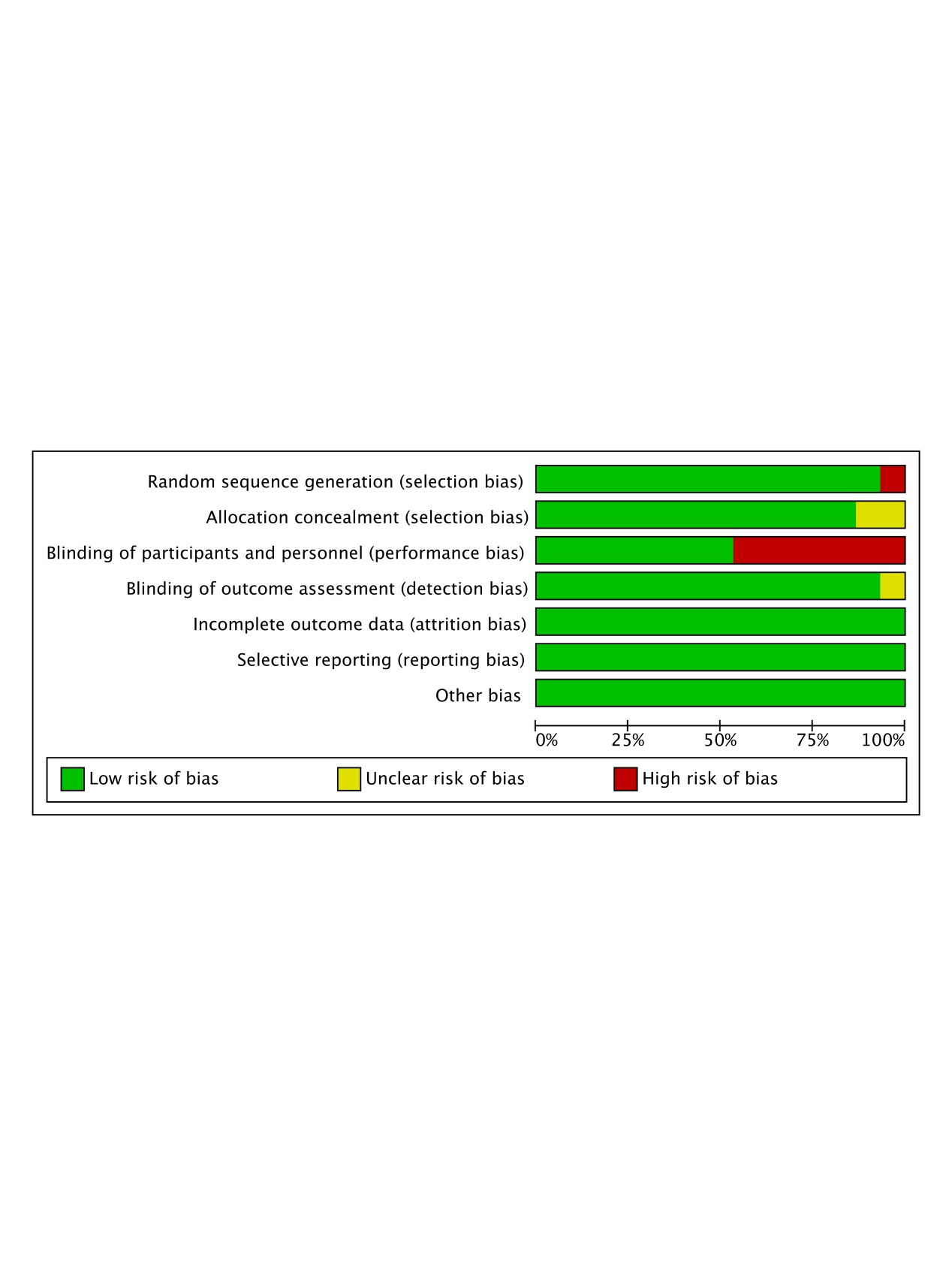
**

Figure S1. Risk of bias graph.

**
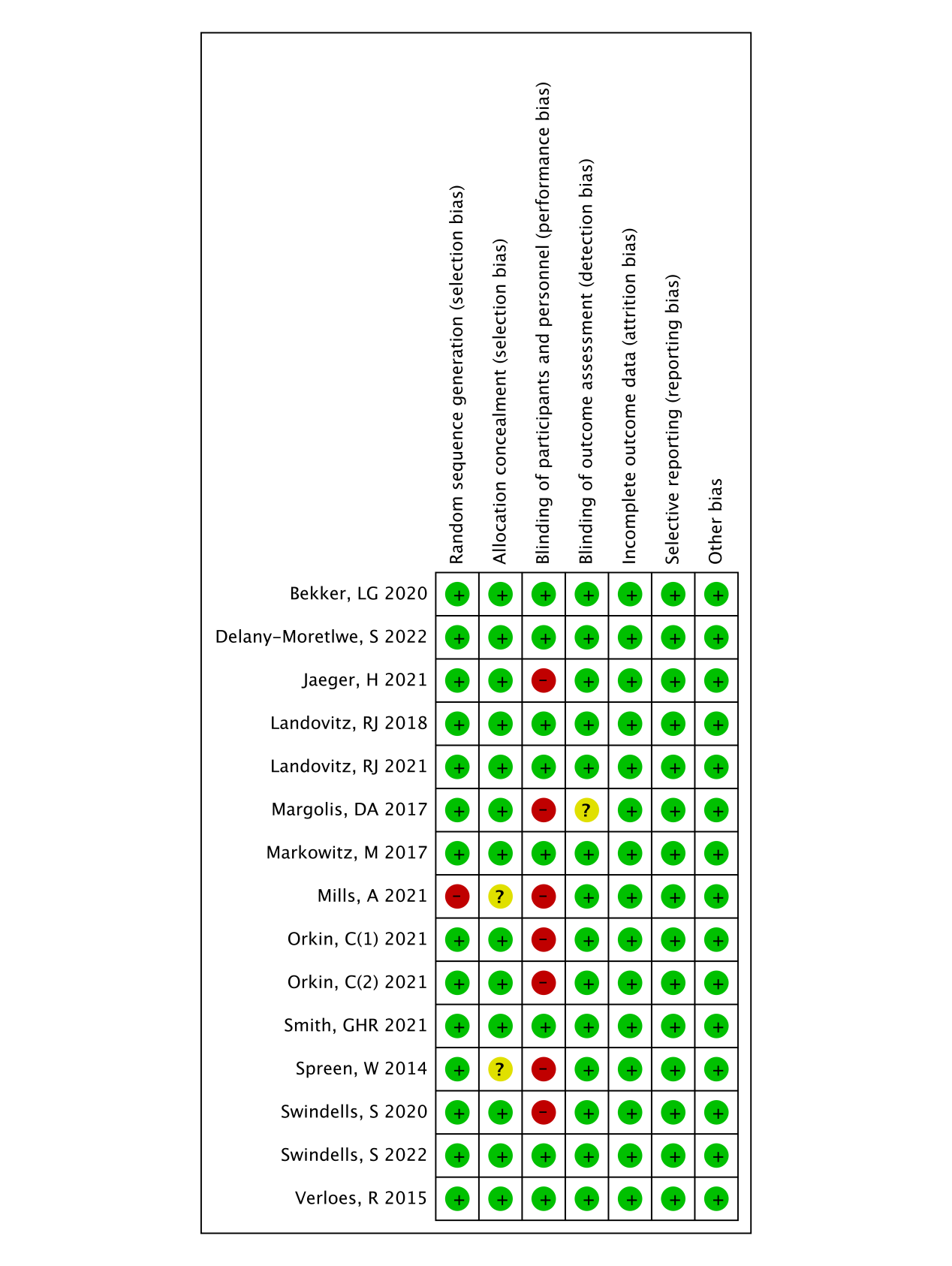
**

Figure S2. Risk of bias summary.
