# Supplementary material for: Safety and Efficacy of Long-Acting Injectable Agents for HIV-1: Systematic Review and Meta-Analysis
Source: JMIR Public Health Surveill. 2023 Jul 27;9:e46767. doi: 10.2196/46767 (PMC10415942; doi:10.2196/46767)
Supplement: Multimedia Appendix 4 [file publichealth_v9i1e46767_app4.docx]

**Multimedia Appendix 4. Funnel plots for the outcomes**

**
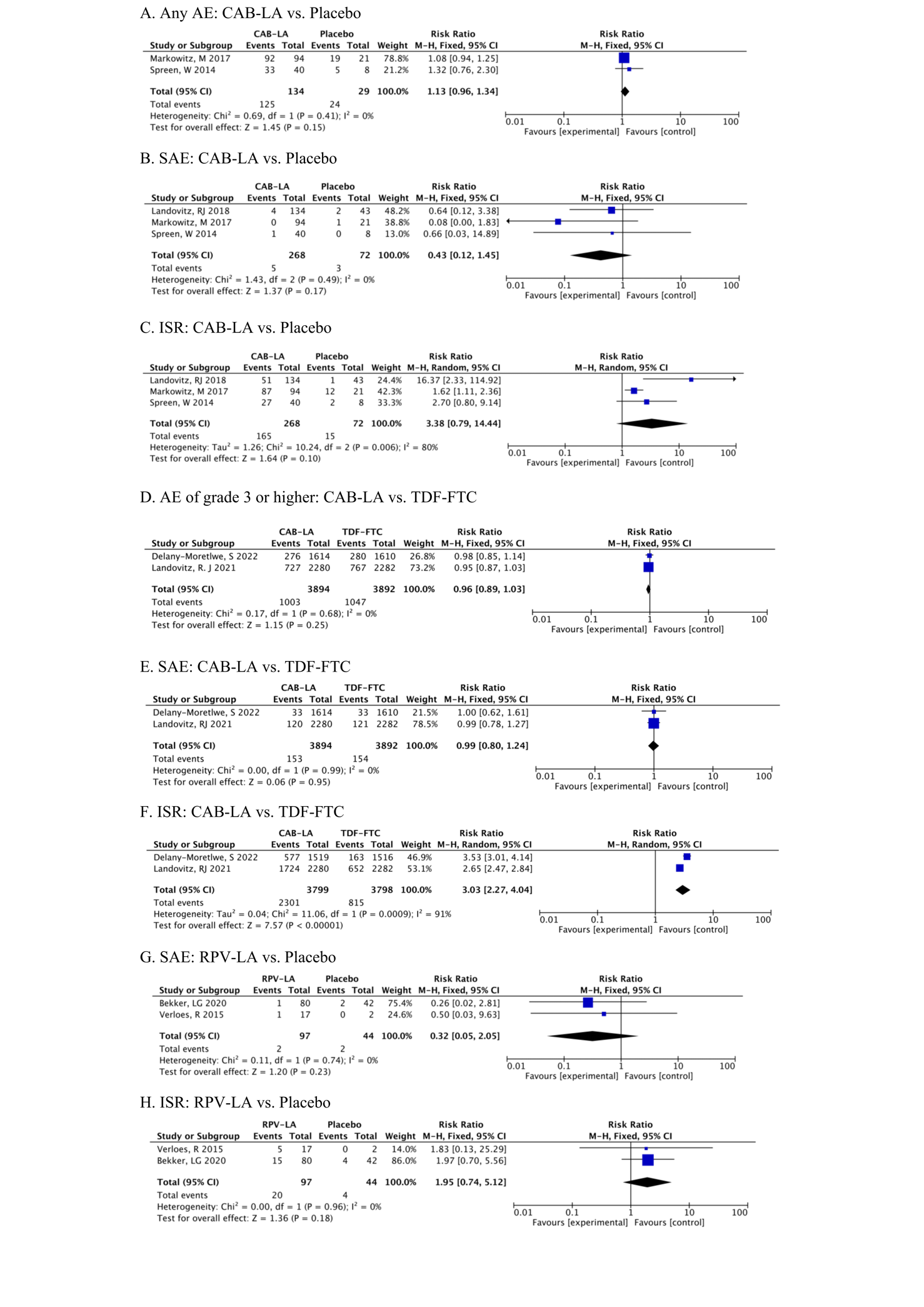
**

Figure S1. Meta-analyses on safety profiles of CAB-LA and RPV-LA.

**
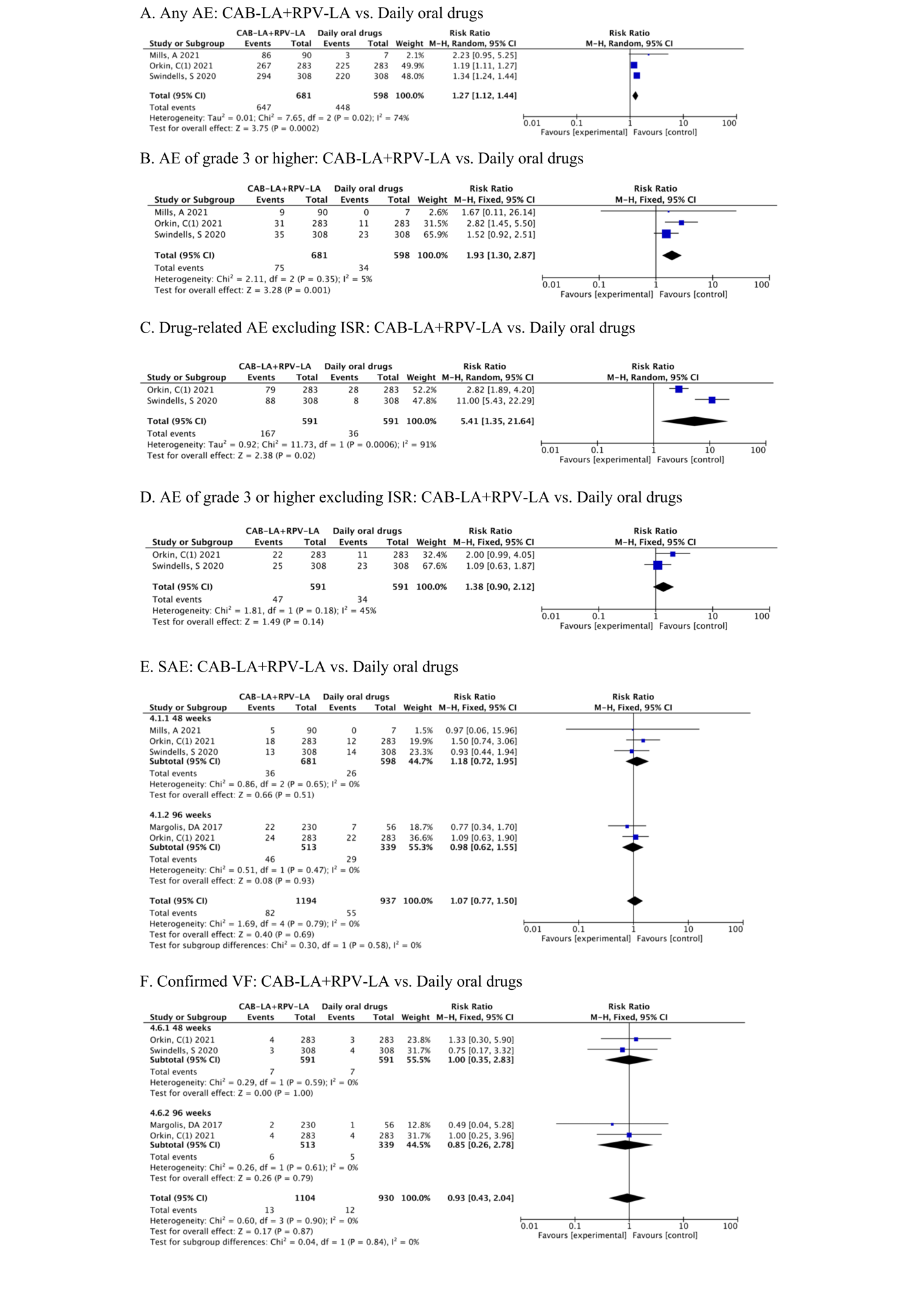
**

Figure S2. Meta-analyses on safety and efficacy profiles of CAB-LA+RPV-LA.

**
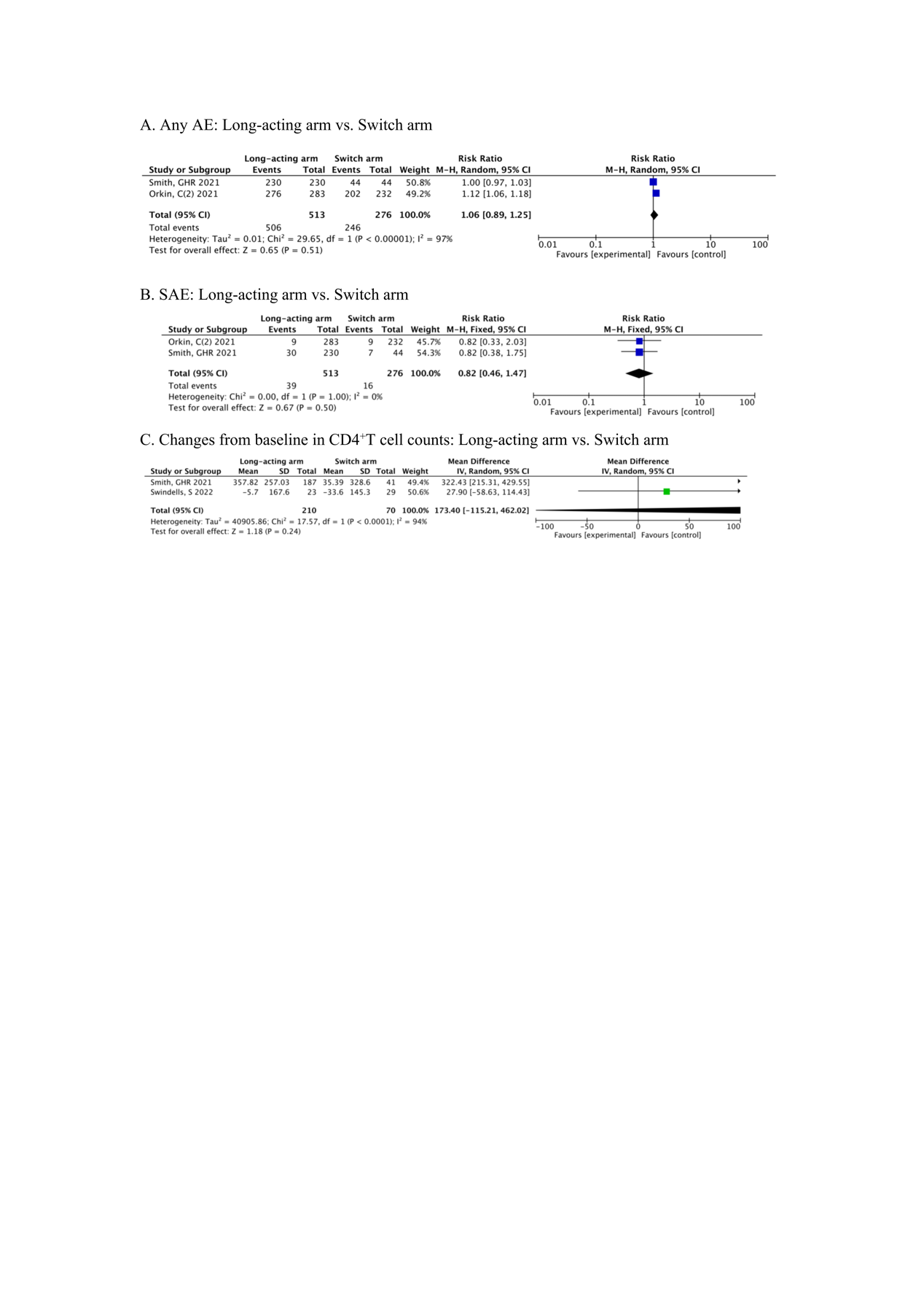
**

Figure S3. Forest plots comparing safety and efficacy between the Long-acting arm and the Switch arm.
