# Supplementary material for: Safety and Efficacy of Long-Acting Injectable Agents for HIV-1: Systematic Review and Meta-Analysis
Source: JMIR Public Health Surveill. 2023 Jul 27;9:e46767. doi: 10.2196/46767 (PMC10415942; doi:10.2196/46767)
Supplement: Multimedia Appendix 5 [file publichealth_v9i1e46767_app5.docx]

**Multimedia Appendix 5. Safety and efficacy profiles of long-acting antiretroviral drugs for prophylaxis^a^**

| Author/Trial name (NCT ID) | Design | Safety | | | | | | | | | |  | Efficacy |  |
| --- | --- | --- | --- | --- | --- | --- | --- | --- | --- | --- | --- | --- | --- | --- |
|  |  | Any AE | Drug-related AE | AE of grade 3 or higher | SAE | | | ISR | | AE withdrawal | Death |  | Confirmed HIV-1 infection |  |
| **CAB-LA** | | | | | | | | | | | |  |  |  |
| Landovitz RJ, et al  HPTN 077  (NCT02178800) | CAB-LA IM 600mg Q8W  CAB-LA IM 800mg Q12W  vs  placebo | NR | NR | NR | | 4/134  2/43 | | 51/134  1/43 | | 10/134  0/43 | 0  0 |  | 1/151  0/48 |  |
| Markowitz M, et al  ECLAIR  (NCT02076178) | CAB-LA IM 800mg Q12W  vs  placebo | 92/94  19/21 | 92/94  15/21 | 19/94  0/21 | | 0/94  1/21 | | 87/94  12/21 | | 4/94  1/21 | 0  0 |  | 1/105  1/21 |  |
| Spreen W, et al  (NCT01593046) | CAB-LA IM 100，200，200×2，400，400×2mg single-dose  vs  placebo | 33/40  5/8 | NR | NR | | 1/40  0/8 | | 27/40  2/8 | | 9/283  4/283 | 0  0 |  | NR |  |
| Landovitz RJ, et al  HPTN 083  (NCT02720094) | CAB-LA IM 600mg Q8w with TDF-FTC placebo QD  vs  TDF-FTC QD with CAB-LA placebo IM Q8w | NR | NR | 727/2280  767/2282 | | 120/2280  121/2282 | | 1724/2280  652/2282 | | NA | 4/2280  7/2282 |  | 13/3205  39/3187 |  |
| Delany-Moretlwe S, et al  HPTN 084  (NCT03164564) | CAB-LA IM 600mg Q8w with TDF-FTC placebo QD  vs  TDF-FTC QD with CAB-LA placebo IM Q8w | NR | NR | 276/1614  280/1610 | | 33/1614  33/1610 | | 577/1519  163/1516 | | 17/1614  23/1610 | 3/1614  0/1610 |  | 4/1956  36/1942 |  |
| **RPV-LA** | | | | | | | | | | | | | | |
| Bekker LG, et al  HPTN 076  (NCT02165202) | RPV-LA IM 1200mg Q8W  vs  placebo | NR | NR | NR | 1/80  2/42 | | 15/80  4/42 | | 6/80  2/42 | | 1/80  0/42 |  | 0/80  1/42 |  |
| Verloes R, et al  (NCT01031589) | RPV-LA IM 300,600 mg single-dose  RPV-LA IM 1200/600/600mg Q4w  vs  placebo | 14/17  1/2 | 8/17  0/2 | NA | 1/17  0/2 | | 5/17  0/2 | | 1/17  0/2 | | NR |  | NR |  |

^a^All results are expressed in terms of frequency(n/N) unless otherwise stated.

**NCT ID,** Clinicaltrials.gov identifier; **CAB,** cabotrgravir; **RPV,** rilpivirine; **TDF,** tenofovir; **FTC,** emtricitabine; **LA,** long acting; **IM,** intramuscular; **QD,** daily; **Q4W,** every 4 weeks; **Q8W****,** every 8 weeks; **Q12W,** every 12 weeks.

**AE,** adverse events; **ISR,** injection-site reaction; **SAE,** serious adverse events; **IM,** intramuscular; **NR,** not reported; **NA,** not available.
