# Supplementary material for: Safety and Efficacy of Long-Acting Injectable Agents for HIV-1: Systematic Review and Meta-Analysis
Source: JMIR Public Health Surveill. 2023 Jul 27;9:e46767. doi: 10.2196/46767 (PMC10415942; doi:10.2196/46767)
Supplement: Multimedia Appendix 6 [file publichealth_v9i1e46767_app6.docx]

**Multimedia Appendix 6. Drug resistance data.**

Table S1. Resistance-associated mutations data

| Author/Trial name (NCT ID) | Design | The proportion of resistance-associated mutations in patients who acquired HIV-1 infection or with confirmed virologic failure | | | |  | Resistance-Associated Mutations | | | |
| --- | --- | --- | --- | --- | --- | --- | --- | --- | --- | --- |
|  |  | INSTI | NNRTI | NRTI | PI |  | INSTI | NNRTI | NRTI | PI |
| **Prophylaxis** | | | | | | | | | | |
| Landovitz RJ,et al  HPTN 077  （NCT02178800） | CAB-LA IM 600mg Q8W  CAB-LA IM 800mg Q12W  vs  placebo | NR | NR | NR | NR |  | NR | NR | NR | NR |
| Markowitz M,et al  ECLAIR  (NCT02076178) | CAB-LA IM 800mg Q12W  vs  placebo | NR | NR | NR | NR |  | NR | NR | NR | NR |
| Spreen W,et al  (NCT01593046) | CAB-LA IM 100，200，200×2，400，400×2mg single-dose  vs  placebo | NR | NR | NR | NR |  | NR | NR | NR | NR |
| Landovitz RJ,et al  HPTN 083  (NCT02720094) | CAB-LA IM 600mg Q8w with TDF-FTC placebo QD  vs  TDF-FTC QD with CAB-LA placebo IM Q8w | 5/16 1/42 | 5/16 10/42 | 1/16 4/42 | 7/16 8/42 |  | E138A, E138K, E138E/K G140A, G140G/S, Q148K, Q148R, R263K  T97A | H221Y, K103N, L100I, P225H, V179T, Y181C  A98G, F227L, H221Y, G190A, G190S, K103N, K103S, P225H, Q207E, T369V, V179D, Y181C | K65R, M184V  K65R, M184I, M184I/V, M184V | A71T, L10I, L10V, L89M, M36I, I62V, K20R  A71T, A71V, D60E, I62V, L10I, L10V, M36I |
| Delany-Moretlwe S, et al  HPTN 084  (NCT03164564) | CAB-LA IM 600mg Q8w with TDF-FTC placebo QD  vs  TDF-FTC QD with CAB-LA placebo IM Q8w | 0/4  NR | NR | NR  1/36 | NR |  | None | NR | M184 V | NR |
| Bekker LG,et al  HPTN 076  (NTC 02165202) | RPV-LA IM 1200mg Q8W  vs  placebo | NR | NR | NR | NR |  | NR | NR | NR | NR |
| Verloes R,et al  (NCT01031589) | RPV-LA IM 300,600 mg single-dose  RPV-LA IM 1200/600/600mg Q4w  vs  placebo | NR | NR | NR | NR |  | NR | NR | NR | NR |
| **Treatment** | | | | | | | | | | |
| Margolis, D. A et al  LATTE-2  (NCT02120352) | CAB-LA IM 400mg + RPV-LA IM 600mg Q4W  CAB-LA IM 600mg + RPV-LA IM 900mg Q8W  CAB PO 30mg + ABC/3TC PO 600/300mg QD | 0  2/2 0/1 | 0  1/2 0/1 | NR | NR |  | None  Q148R, R269 R/G  None | None  E138G, K103N, K238T  None | NR | NR |
| Smith GHR et al  LATTE-2 (extension phase)  (NCT02120352) | CAB-LA IM 400mg + RPV-LA IM 600mg Q4W  CAB-LA IM 600mg + RPV-LA IM 900mg Q8W  Optimized loading dose (100w) followed by CAB-LA IM 400mg + RPV-LA IM 600mg Q4W  Optimized loading dose (100w and 104w) followed by CAB-LA IM 600mg + RPV-LA IM 900mg Q8W | NR | NR | NR | NR |  | NR | NR | NR | NR |
| Orkin, C et al  FLAIR  (NCT02938520) | CAB-LA IM 400mg + RPV-LA IM 600mg Q4W  DTG/ABC/3TC PO 50/600/300mg QD | 3/4 0/4 | 3/4 1/4 | NR | NR |  | G140R, Q148R  None | E138E/A/K/T, E138K, K101E  V179V/I | NR | NR |
| Orkin, C et al  FLAIR (extension phase)  (NCT02938520) | DTI group (after 24w of CAB + RPV)  OLI group (after 24w of CAB + RPV)  Randomly assigned long-acting arm (after 124w of CAB + RPV) | 0/1  0  4/5 | 0/1  0  4/5 | NR | NR |  | None  A155H, A263L, G140R, Q148R | None  E138E/A/K/T, E138K, G138G, K101E, M230L, V106V/A, V108V/I | NR | NR |
| Swindells, S et al  ATLAS  (NCT02951052) | CAB-LA IM 400mg + RPV-LA IM 600mg Q4W  PI-, NNRTI-, or INSTI-based QD | 1/3 0/4 | 3/3 2/4 | 0/3 2/4 | 1/3 0/4 |  | N155H  None | E138A, E138E/K, E138K, V108I  G190S, M230M/I | None  M184I, M184V | N88N/S  None |
| Swindells, S et al  ATLAS（extension phase）  (NCT02951052) | CAB-LA IM 400mg + RPV-LA IM 600mg Q4W  Switched from CAB 30mg + RPV 25mg QD to CAB-LA IM 400mg +RPV-LA IM 600mg Q4W | NR | NR | NR | NR |  | NR | NR | NR | NR |
| Jaeger H, et al  ATLAS-2M  (NCT03299049) | CAB-LA IM 400mg +RPV-LA IM 600mg Q4W  CAB-LA IM 600mg +RPV-LA IM 900mg Q8W | 2/2 5/9 | 2/2 6/9 | NR | NR |  | N155N/H, Q148R  N155N/H, N155H, Q148Q/R, Q148R, T97A | E138E/K, K101E, M230L  E138A, E138E/K, K101E, K103N, P225H, Y188L | NR | NR |
| Mills A, et al  POLAR  (NCT03639311) | CAB-LA IM 600mg +RPV-LA IM 900mg Q8W  DTG/RPV 50/25mg QD | NR | NR | NR | NR |  | NR | NR | NR | NR |

^a^All results are expressed in terms of frequency(n/N) unless otherwise stated.

**NCT ID,** Clinicaltrials.gov identifier; **CAB,** cabotrgravir; **RPV,** rilpivirine; **TDF,** tenofovir; **FTC,** emtricitabine; **LA,** long acting; **IM,** intramuscular; **QD,** daily; **Q4W,** every 4 weeks; **Q8W****,** every 8 weeks; **Q12W,** every 12 weeks.

**INSTI,** integrase inhibitor; **NNRTI,** nonnucleoside reverse transcriptase inhibitor; **NRTI,** nucleoside reverse transcriptase inhibitor; **PI,** protease inhibitor; **NR,** not reported.


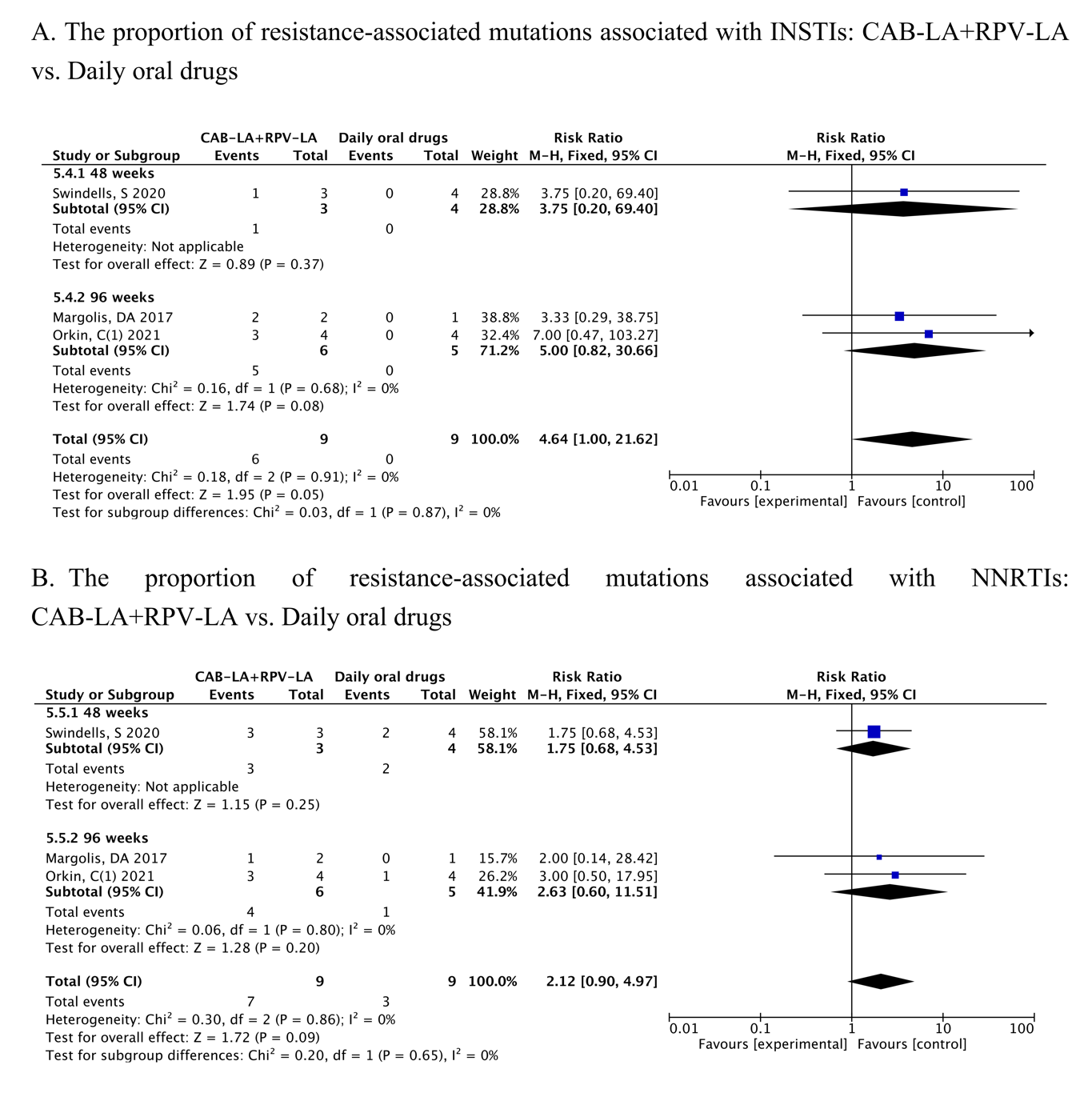


Figure S1. Meta-analyses on drug resistance profiles of CAB-LA+RPV-LA


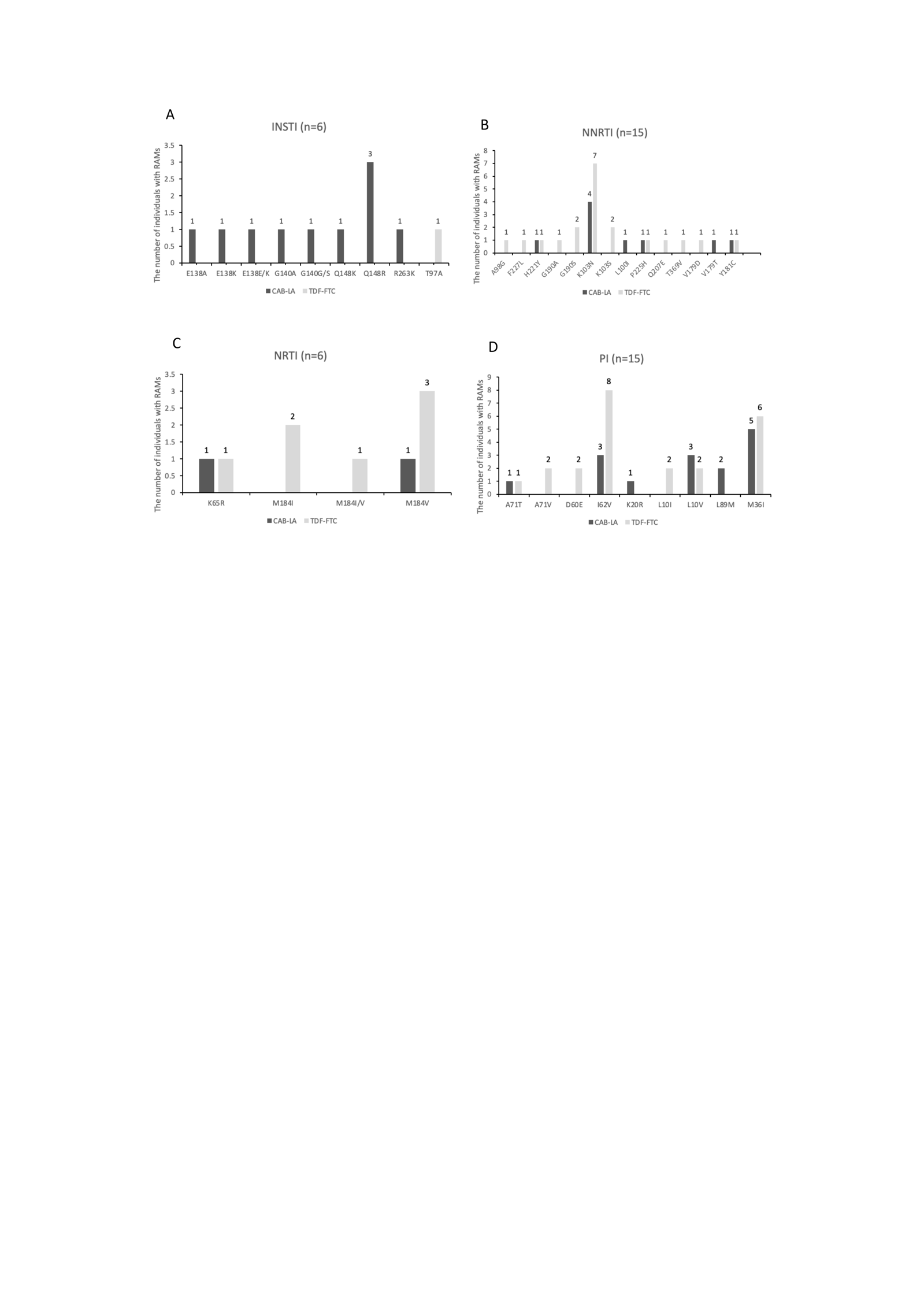


Figure S2. Bar charts of the number of individuals with RAMs: CAB-LA vs. TDF-FTC


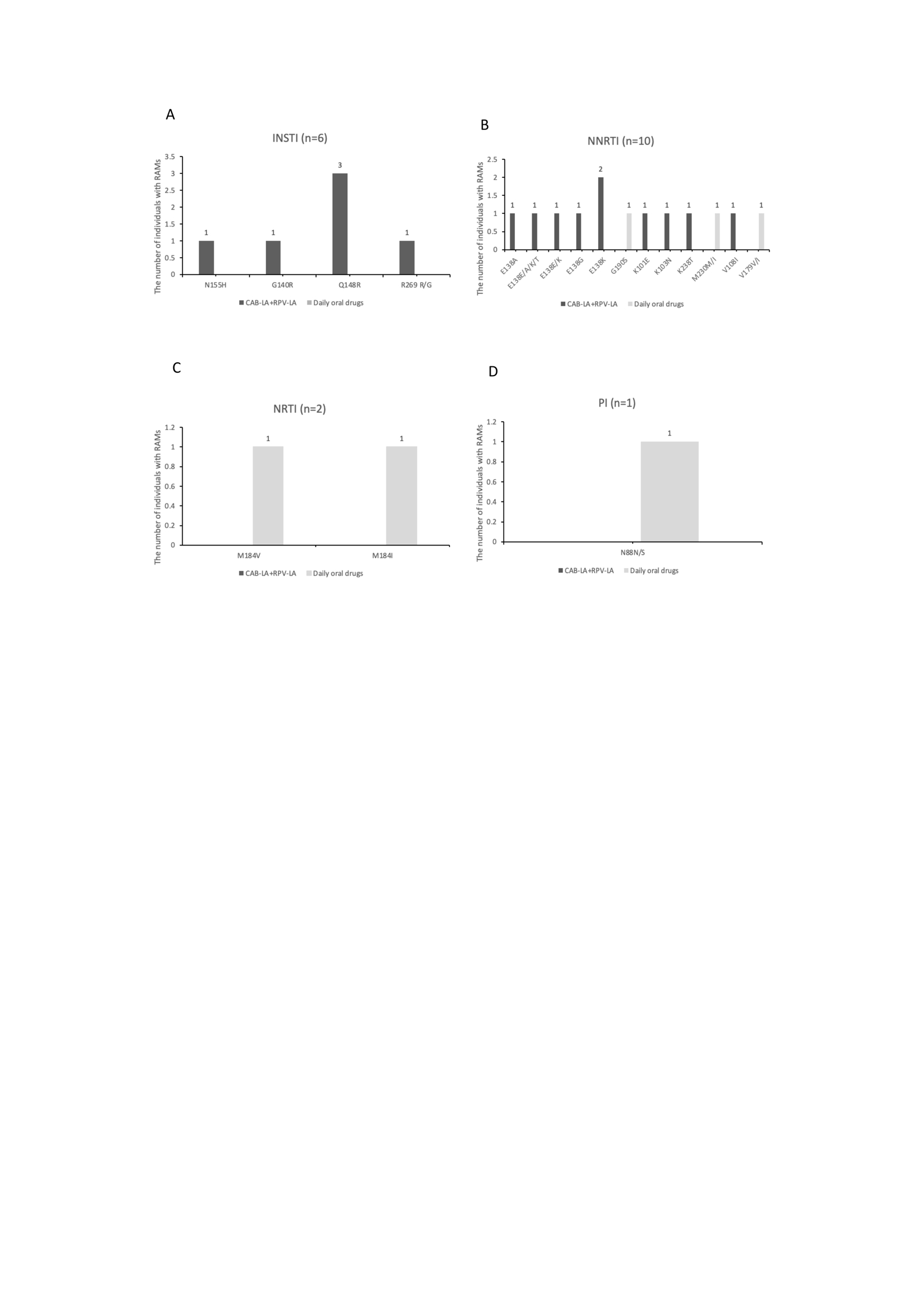


Figure S3. Bar charts of the number of individuals with RAMs: CAB-LA+RPV-LA vs. Daily oral drugs
